# Research protocol of the efficacy of probiotics for the treatment of alcohol use disorder among adult males: A comparison with placebo and acceptance and commitment therapy in a randomized controlled trial

**DOI:** 10.1371/journal.pone.0294768

**Published:** 2023-12-05

**Authors:** Bingyu Zhang, Ruiling Zhang, Hongdu Deng, Ping Cui, Chunyan Li, Fan Yang, Mohammad Farris Iman Leong Bin Abdullah

**Affiliations:** 1 Department of Community Health, Advanced Medical and Dental Institute, Universiti Sains Malaysia, Kepala Batas, Pulau Pinang, Malaysia; 2 Department of Psychiatry, 2^nd^ Affiliated Hospital, Xinxiang Medical University, Xinxiang, Henan, China; University of Minho, PORTUGAL

## Abstract

**Background and aim:**

Primarily, this study compares the efficacy of probiotic and acceptance and commitment therapy (ACT) in alleviating the severity of alcohol craving and alcohol use disorder (AUD) among patients who had undergo two weeks of in-patient detoxification. Secondarily, this study compares the efficacy of probiotic and ACT in mitigating the severity of comorbid depression and anxiety symptoms; decreasing serum level of pro-inflammatory cytokines, such as interleukin 1β (IL-1β), interleukin 6 (IL-6), and tumor necrosis factor α (TNF-α); changing the event-related potential in electroencephalogram (EEG) and restoring microbiota flora in the gut of AUD patients.

**Methods and analysis:**

Initially, during Phase I of the study, the serum level of IL-1β, IL-6 and TNF-α; ERP changes in the EEG and fecal microbiota content will be compared between 120 AUD patients and 120 healthy controls. Subsequently in Phase II of the study, 120 AUD patients will be randomized by stratified permuted block randomization into the probiotic, ACT and placebo groups in a 1:1:1 ratio. Participants in the probiotic and placebo groups will be administered one sachet per day of *Lactobacillus spp*. probiotic and placebo, respectively for 12 weeks. While those in the ACT group will receive one session per week of ACT for 8 weeks. Outcome measures will be administered at four timepoints, such as t_0_ = baseline assessment prior to intervention, t_1_ = 8 weeks after intervention began, t_2_ = 12 weeks after intervention and t_3_ = 24 weeks after intervention. Primary outcomes are the degrees of alcohol craving, alcohol withdrawal during abstinence and AUD. Secondary outcomes to be assessed are the severity of co-morbid depression and anxiety symptoms; the serum levels of IL-1β, IL-6 and TNF-α; changes in ERP and fecal microbiota content.

**Trial registration number:**

NCT05830708 (ClinicalTrials.gov). Registered on April 25, 2023.

## Introduction

Alcohol is a common and easily accessible addictive substance. There are a large number of drinkers worldwide, and the impact of alcohol-related problems cannot be ignored. According to the latest Global Status Report on Alcohol and Health 2018 released by the World Health Organization (WHO), there are about 2.3 billion drinkers in the world at present. In 2016 alone, about 3 million deaths could be attributed to the harmful use of alcohol, accounting for 5.3% of the total number of deaths, while drinking was also responsible for 132.6 million disability-adjusted life years (DALYs) [1]. Alcohol consumption is related to more than 60 types of diseases and injuries, and major health issue associated with alcohol use is alcohol use disorder (AUD) [2]. The prevalence of AUD in China is also increasing year by year. At present, the prevalence of AUD is 6.9% in males and 0.2% in females [3].

The core symptoms of AUD mainly include two aspects: one is physical dependence based on increased alcohol tolerance and withdrawal symptoms, and the other is psychological dependence based on alcohol craving [4]. Currently, clinical treatment for AUD focus on reducing physical dependence. Benzodiazepines (BZD) are usually given to alleviate withdrawal symptoms, low-dose antipsychotics to relieve psychotic symptoms, and adequate vitamin B supplementation to prevent Wernicke encephalopathy. However, most patients still have a strong craving for alcohol during abstinence, even after receiving standardized in-patient treatment. This psychological dependence on alcohol is hard to resist, so it is extremely difficult to maintain long-term abstinence, and AUD is prone to relapse [5, 6]. Therefore, psychological dependence is the key factor leading to people start drinking again after abstinence, and the monitoring and treatment of alcohol craving is an important link to improve the therapeutic effect of AUD [6].

### Event-related potential as biomarker in alcohol use disorder

One way to monitor alcohol craving among patients with AUD is by assessing the event-related potential (ERP) under electroencephalogram (EEG) monitoring. ERP is a special evoked EEG. When the body receives multiple or diverse events with psychological significance, there will be a "time-locked" relationship between the EEG and the stimulation, which can reflect the neural electrophysiological phenomenon of the brain in the process of cognitive processing. ERP is a response in the brain in the form of electrical activity which is measured by EEG and this response arise after occurrence of specific events which could be sensory, motor or cognitive experience [7]. Some studies suggest that abnormal ERP amplitude may be a disease marker of substance dependence and a potential neurobiological endophenotype [8]. Bartholow et al. (2010) used an experimental paradigm containing images with alcohol cues to detect P300 and found that the P300 response of subjects with low sensitivity to alcohol was significantly enhanced, while that of subjects with high sensitivity to alcohol was not [9].

Moreover, such enhanced P300 response was alcohol-specific and would not be extended to other motivational related stimuli. Alcohol cue-induced P300 may be a sensitive endophenotype for the risk of alcohol addiction [9]. Petit et al. (2013) reported that alcoholics had enhanced ERP responsiveness to alcohol-related cues, especially male patients [10]. A previous study found that there were positive findings in EEG analysis of AUD patients in the craving state induced by static pictures of alcohol cues, in which AUD patients presented with lower N200 amplitude and higher P300 amplitude under the stimulation of alcohol cues, which were positively correlated with the subjective craving scale score [3]. Hence, it may be possible to objectively assess the psychological dependence of AUD patients by using alcohol cue response to induce the craving for alcohol and monitoring the task-state EEG of the subjects’ ERP at the same time. However, further studies are warranted to confirm the implication of ERP in the assessment of psychological dependence of AUD patients by determining how ERP would response to effective treatment of alcohol craving.

### Relationship between intestinal microbiota, probiotic, and alcohol dependence

Over the past decade, the link between the gut, and specifically the gut microbiome, and alcohol dependence has come to the attention of researchers. In individuals with alcohol addiction, drinking can disrupt intestinal barrier function, also known as intestinal leakage [11, 12]. The intestinal barrier consists of intestinal cells, goblet cells, and antibacterial substances that influence the intestinal microbiota within the mucus layer, as well as many immune cells in the lamina propria [13]. The mechanism by which alcohol or its metabolites cause intestinal leakage is unknown, and may be related to intestinal disorders, immune system activation, and inflammation. Persistent alcohol abuse can change fecal pH, promote excessive growth of pathogens, and alter the function of intestinal microorganisms by altering the secretion of specific metabolites involved in intestinal barrier dysfunction [14, 15]. Elevated levels of plasma cytokines such as TNF α, interleukin and CRP in patients with AUD suggest chronic, low-grade, systemic inflammation [16]. One possible mechanism of systemic inflammation and alcohol addiction is that intestinal bacterial products activate peripheral blood monocytes and induce cytokines to enter the blood, leading to alcohol addiction and other psychiatric disorders, including major depressive disorder, bipolar disorder and anxiety disorder [17]. Alcohol cravings and negative emotions such as anxiety and depression were highly associated with alcohol-seeking behavior and relapse, suggesting that reducing systemic inflammation may improve mental health and prevent relapse [17].

#### Probiotics hold promise for treating alcohol dependence

Probiotics are healthy microbes, and alcohol dependent patients had fewer bifidobacteria and lactobacillus in their gut than the normal population [13]. As mentioned above, altered gut barrier function affects alcohol-related behaviors in alcohol-dependent patients, and the gut barrier is regulated by specific bacteria [18–21], transplantation of specific gut microbes into the gut of alcohol-dependent patients can play a positive role in the treatment of alcohol-dependent patients. As in a double blind randomized controlled trial, consumption of Lachnospiraceae and Ruminococcaceae-containing probiotic confers significant increase in gut microbiota diversity, a significant decrease in alcohol craving and drinking, and a significant decrease in alcohol abuse-related behaviors among subjects in the test group compared to subjects in control group [19]. This suggests that gut microbiota may be a potential treatment for alcohol dependence.

Temko et al. (2017) reported that alcohol dependence, along with other substance use disorders and eating disorders are associated with alterations in the brain’s "reward" pathways [20], and changes in gut microbiota may alter biological pathways by reducing systemic inflammation. Probiotics such as Lactobacillus, Bifidobacterium, and Ackermannia may enhance intestinal integrity through a variety of mechanisms [21–24], normalizing intestinal cytokine levels, balancing intestinal immunity, and reducing inflammation. In a clinical study by Vatsalya et al. (2023), 24 subjects who consumed Lactobacillus rhamnosus GG(LGG) for 180 days showed a significant reduction in severe alcohol consumption and even achieved abstinence from alcohol compared to 22 subjects on placebo. In addition to this, subjects taking LGG had significantly better liver function than those consuming placebo. Therefore, the use of probiotics in the treatment of alcohol dependence and substance dependence may be a promising treatment [25].

In addition, several clinical studies reported that probiotics have a role in modulating mood and social functioning [26]. In clinical study which examined the efficacy of Rosell^®^-52 and Rosell^®^-175 (3×10^9^ CFU of probiotics daily), after four weeks of probiotic consumption, functional magnetic resonant imaging registered a reduction in the activation of the lateral orbital and ventral cingulate regions and a significant increase in functional connectivity between the superior limbic and mid-ventral regions were observed compared with those on placebo. This suggests that the Rosell^®^-52 and Rosell^®^-175 interventions were able to alter functional connectivity in regions of the brain that are active and regulate emotional and stress responses. This result is a more intuitive demonstration of the ability of probiotics to improve brain function and emotional responses via the gut-brain axis [27]. In addition, Rosell^®^-52 and Rosell^®^-175 (daily intake of 10^10^ CFU for 30 days) showed better results in alleviating depressed mood and pro-inflammatory cytokines among patients with depression compared with control subjects on placebo [28]. This suggests that probiotic administration may improve depressive symptoms through the gut-brain axis. Another double-blind randomized controlled trial also demonstrated that significant improvement in depression and anxiety symptoms as well as attenuation of cytokine IL-1α and IL-6 were observed in subjects on probiotics (daily intake for 30 days) compared with controls on placebo [29]. This suggests that probiotic administration may also improve depression with comorbid anxiety symptoms through the gut-brain axis. These data suggest that probiotics have beneficial effects on the enteric-liver axis in alcohol-dependent patients.

### Acceptance and commitment therapy (ACT)

ACT is a third-generation therapy, which is new from cognitive behavioral therapy (CBT), and it is approach to produce psychological flexibility [30]. ACT was designed to increase adaptive coping through acceptance, cognitive defusion, mindfulness, and perspective-taking exercises while supporting clients in aligning behavior with their personal values [31, 32]. ACT facilitates development and maintenance of health behavioral improvements by targeting internal barriers [33]. ACT guides people with SUD to accept the urges for substance and the symptoms and applied psychological flexibility and value-based intervention to ameliorate urges and symptoms. However, data on the efficacy of ACT for treatment of AUD is lacking and further exploration on the effects of ACT is warranted.

### Rationale to conduct this study

Although, current treatment is available for detoxification of patients with AUD to resolve alcohol withdrawal, relapse among AUD patients remain high due to the presence of psychological dependence such as craving. The efficacy of currently available treatment for alcohol use disorder have limited success to manage alcohol craving, ameliorate the severity of AUD, and prevent relapse. Probiotic supplement have shown some promise in the treatment of AUD as restoration of the gut microbiota may reduce systemic inflammation, increase brain derived neurotrophic factor and glutamate receptors in the hippocampus [34]. In addition, novel psychotherapy such as ACT may also help AUD patient to accept and control urge for alcohol and hence may be effective to reduce alcohol craving and prevent relapse. In essence, it will be interesting to conduct a comprehensive randomized controlled trial to compare the efficacy of probiotic supplement (experimental intervention) and ACT (active comparator) in treatment of AUD, preventing alcohol craving, and treating comorbid depression and anxiety symptoms.

### Objective

#### Primary objective

To determine the differences in the severity of alcohol use disorder, alcohol withdrawal and craving among patients on Lactobacillus sp., placebo and ACT, at 4 timelines (baseline, 8 weeks after starting intervention, 12 weeks after starting intervention, and 24 weeks after starting intervention which is 12 weeks post termination of intervention).

#### Secondary objectives

1. To determine the differences in the serum levels of pro-inflammatory cytokines among patients on Lactobacillus sp., placebo and ACT, at 4 timelines (baseline, 8 weeks after starting intervention, 12 weeks after starting intervention, and 24 weeks after starting intervention which is 12 weeks post termination of intervention).
2. To assess the differences in severity of depression and anxiety symptoms among patients on Lactobacillus sp., placebo and ACT via use of questionnaire, at 4 timelines (baseline, 8 weeks after starting intervention, 12 weeks after starting intervention, and 24 weeks after starting intervention which is 12 weeks post termination of intervention).
3. To investigate the changes of ERP (evoked by alcohol cues related task) characteristics in patients with AD after the above related treatments, at 3 timelines (baseline, 8 weeks after starting intervention, 12 weeks after starting intervention).
4. To assess the differences in gut microbiota profiles of patients on Lactobacillus sp., placebo and ACT via the use of fecal samples, at 3 timelines (baseline, 8 weeks after starting intervention, and 12 weeks after starting intervention).

## Materials and methods

### Research design

Phase I of the study is a case-control study where the case and control subjects are age, gender and ethnicity matched. Then, Phase II of the study is a single-blind, three-armed, parallel-group, multi-center randomized controlled trial. The entire study will be conducted for a duration of three years.

### Study area

The study will be conducted in the Second Affiliated Hospital of Xinxiang Medical University (Henan Mental Hospital), Xinxiang, Henan, China and Psychiatric Outpatient Clinic in Advanced Medical and Dental Institute, Universiti Sains Malaysia.

### Ethical consideration

The approval of the study was obtained from the Human Research Ethics Committee of Xinxiang Medical University and from the Human Research Ethics Committee of Universiti Sains Malaysia. The study will be conducted according to the Declaration of Helsinki 1974 and its subsequent amendments and abide by the Good Clinical Practice Guidelines for Clinical Trial in Malaysia. The research protocol approved by the Human Research Ethics Committee of Xinxiang Medical University and the Human Research Ethics Committee of Universiti Sains Malaysia is included as S1 Appendix. If there is any amendments, for example in the eligibility criteria, study population, study procedures, interventions, data collection, data analysis; it will be informed in writing to the Human Research Ethics Committee of Xinxiang Medical University and the Human Research Ethics Committee of Universiti Sains Malaysia.

Prior to participation, the rights of the participants are explained by the research assistant. Participants anonymity will be assured. The personal data of the participants will not be used for reporting of findings and publication. Instead, group data will be used for reporting and publication of findings. Each participant will be assigned specific research code for identification purposes rather than using personal identifiable information, for example RCT001, RCT002, etc. The data collected from participants will be stored in the research team laptop and/or thumb drive which will be locked in a file cabinet at the research team office with the keys kept by the principal investigator. The data collected from the participants is accessible only by the principal investigator and the co-researchers. The data collected from the participants will not be documented in the patient case file of the particpants. Prior to participation, all the participants will also be informed regarding the study purposes, study procedures, and the risks and benefits of participating in the study. The English version of the Participant Information Sheet and Consent Form are included in S2 Appendix.

The findings of the study will be published in academic journals and presented in conferences or symposia. As for publication of research findings in academic journals, the principal investigator will be the corresponding author while the other authorship will be determined according to the International Committee of Medical Journal Editors’ recommendations. All the investigators in the research team declared that there is no competing interest in term of financial gain or the conduct of this study.

Participants who experienced any adverse effects (AE) will be withdrawn from the study. AE is any untoward medical events which occurred with or without causal relationship with the study’s intervention. Any AE which occurred among the participants will be monitored by using the clinical trial card which contains the name of the participant, address, date and time of the AE, description of the AE, cause of the AE, any treatment given for the AE, and duration of the AE. Participants is expected to report any AE to the principal investigator and the occurrence of AE will be recorded in the clinical trial case report form. The details of the AE reporting will contain information, such as the name of the AE, the date of occurrence and date of resolution, severity, AE relationship with the study intervention, treatment of AE, and the outcome (resolve or still on-going). Participants may withdraw from the clinical trial if there is occurrence of: (1) AE which causes discomfort to the participant although it may not be related to the intervention of the trial, (2) AE which is related to the intervention of the trial and (3) AE which causes change in temperament or personality, suicidal tendency and/or psychotic symptoms after the intervention started. Any occurrence of AE will be reported to the Human Research Ethics Committee of Xinxiang Medical University and Universiti Sains Malaysia before an investigation on the AE is conducted. If there is any safety concerns regarding the administration of the study intervention (probiotic, ACT and/or placebo), such as deliberate self-harm, suicidal tendency, bacterial infection, sepsis, hospitalization due to psychological adverse effect arise from the intervention, or mortality; there it is an indication for premature termination of the trial after an interim analysis. Interim analysis will be carried out by the clinical trial coordination unit of 2^nd^ Affiliated Hospital of Xinxiang Medical University and Advanced Medical and Dental Institute, Universiti Sains Malaysia. During post-trial, those who have serious AE will be referred to the nearest treatment facility for treatment and compensation will be provided when the serious AE is related to the interventions administered in the study.

A trial center will be established and led by the principal investigator who will closely coordinate with the site and research coordinator to monitor the day-to-day conduct of the clinical trial, to recruit participants for the trial and for data collection. A trial monitoring unit will be established and headed by the principal investigator, in which weekly meetings will be held to monitor the conduct of the trial, to prepare for reporting of the trial and auditing of the trial. A trial audit unit will be set up by the Human Research Ethics Committee of Xinxiang Medical University and Universiti Sains Malaysia to monitor the trial data independently from the trial funder. In addition, an interim analysis will be conducted by the trial audit unit if necessary to assess the trial data and if required, the trial may be terminated prematurely based on the report of the interim analysis.

### Phase I of the study

During Phase I of the study, the source population for the experimental subjects is from patients with alcohol-related mental illness who are registered under the Addiction Psychiatry out-patient clinic of the 2^nd^ Affiliated Hospital of Xinxiang Medical University and the Psychiatric out-patient clinic of Advanced Medical and Dental Institute, Universiti Sains Malaysia. While the source population for the healthy control subjects is from the family members of the patients with alcohol-related disorders. In order to ensure that sufficient number of participants are recruited to meet the estimated sample size, advertisement for study participation are posted in both the targeted centers for subject recruitment and honorarium will be provided for participants to compensate for their travelling expenses.

The estimated sample size required for Phase I of the study which compares the serum pro-inflammatory cytokines (IL-1β, IL-6 and TNF-α), the event-related potential in EEG and the fecal microbiota content between the experimental group (patients diagnosed with AUD) and the healthy control group was estimated using the G*Power 3.1.9.7 calculator for estimating sample size needed to compared between two independent means: the α error was 0.05, the power was 0.8, the allocation ratio was 1:1 and the effect size was 0.4 [35]. The calculated sample size required for Phase I was 120 subjects per group (which included 20% of drop out rate).

#### Inclusion and exclusion criteria for experimental subjects

Patient will be included as experimental subjects if: (1) hospitalized patients diagnosed with untreated alcohol use disorder (confirmed by the diagnostic criteria of DSM-5), (2) male gender, (3) age between 18 to 55 years old, (4) Alcohol Use Disorder Identification Test (AUDIT) score of ≥ 8, (5) Han ethnicity, (6) education attainment up to junior high school and above, (7) right-handed person, and (8) has normal eyesight, including corrected vision (patient will be screened clinically).

However, patient will be excluded if they fulfill any of the exclusion criteria include: (1) having history of other mental illnesses (patients will be screened by DSM-V criteria), (2) history of allergy reaction to medications use for conventional treatment of alcohol use disorder, (3) history of past and current medical illnesses which could affect EEG findings, such as organic brain disease, pacemaker implant, gastrointestinal surgery, or serious health problems, (4) had history of seizures, (5) history of illicit drug use (except for nicotine use or dependence), (6) history of taking medications affecting intestinal microbiota 30 days before and during admission (such as antibiotics, omeprazole, ACE inhibitors, alpha blockers, angiotensin-II-receptor antagonists, antihistamines such as H1 inhibitor, beta blockers, calcium, laxatives, metformin, opioids, platelet agregation inhibitors such as aspirin, selective serotonin reuptake inhibitors, statins, tricyclic antidepressant and vitamin D) [36], (7) history of participation in any other alcohol-related studies or trials within the past 30 days, (8) history of using any prescription or over-the-counter drugs in the past 30 days that may affect mood or alcohol cravings, and (9) history of participation in any psychotherapy for treatment of AUD and/or other psychiatric illnesses.

#### Inclusion and exclusion criteria for healthy controls

The inclusion criteria for healthy controls are similar to that for experimental subjects except they must not be diagnosed with alcohol use disorder with DSM-5, must be free from intake of any alcoholic beverage for 2 weeks prior to participation in study, and taking only conventional standard consumption of alcohol (according to World Health Organization definition of average intake of pure alcohol per week is less than 210 grams) or do not drink at all. The exclusion criteria of the healthy controls are also similar to that of experimental subjects.

#### Dropout criteria

The dropout criteria of the subjects include: (1) subjects who withdraw consent for participation, (2) subjects who are non-compliance to intervention or are lost to follow up, (3) subjects who experience severe AE and (4) subjects where the clinical trial cannot be completed due to other reason(s).

#### Recruitment of subjects

The sampling method use for recruitment of patient is consecutive sampling in Phase I of the study. Advertisement will be posted in the notice boards of the targeted centres for subject recruitment and honorarium will be offered to compensate for the transportation to the targeted centres and effort spent to participate in this study. Initially, potential patients registered for treatment for alcohol related disorder in 2^nd^ Affiliated Hospital of Xinxiang Medical University and Psychiatry Outpatient Clinic of Advanced Medical and Dental Institute, Universiti Sains Malaysia will be approached by the research assistants and explained about the study objectives and procedures, to recruit for the experimental group subjects. The control group subjects will be recruited from the family members of the registered patients who are age, gender and ethnicity matched compared with the experimental group subjects. Then, they will be screened for the inclusion and exclusion criteria to classify them into experimental and control subjects during Phase I of the study. All eligible patients will be invited to participate in the study. Those who voluntarily agree to participate in the study will be explained regarding their rights to withdraw from the study at any time and their data collected will be discarded and not use for the study. They will also be explained regarding the benefits and risks of participating in the study, ensure anonymity of personal data and given a copy of the informed consent to sign. They will be given 48 hours to decide on participation in the study and sign the informed consent before they are enroll in the study.

#### Data collection

Phase I of this study aims to compare the differences in serum pro-inflammatory cytokines (TNF- α, IL-1β, and IL-6), electroencephalogram (EEG) changes, and fecal microbiota content between patients diagnosed with AUD (experimental group subjects) and healthy control subjects. Initially, routine treatment for patients diagnosed with alcohol use disorder will be carried out during the first 2 weeks of hospitalization. Detoxification treatment was carried out at the early stage of admission, mainly with benzodiazepine replacement therapy, while adequate supplementation of vitamin B_6_, strengthening symptomatic supportive treatment, and corresponding antipsychotic drugs, antidepressants or mood stabilizers were given according to the condition. Benzodiazepines are gradually discontinued after withdrawal symptoms disappear. Then, after 2 weeks of hospitalization whereby the alcohol withdrawal symptoms have remitted and before they discharge from the hospital, the experimental group subjects (patients with alcohol use disorder) and healthy controls undergo baseline assessment. During baseline assessment, the alcohol use disorder subjects are administered the socio-demographic and clinical characteristics questionnaire, the Chinese versions of the Alcohol Use Disorder Identification Test (AUDIT), the Clinical Institute Withdrawal Assessment for Alcohol-Revised (CIWA-Ar), Penn Alcohol Craving Scale (PACS), Hamilton Depression Rating Scale (HAM-D), and Hamilton Anxiety Rating Scale (HAM-A). In addition, event-related potential (ERP) detection in electroencephalogram (EEG), blood sample collection for pro-inflammatory cytokines (TNF- α, IL-1β, and IL-6), and fecal sample collection for microbiota analysis will also be carried out from the experimental group subjects. As comparison, the healthy control subjects will be administered the socio-demographic and clinical characteristics questionnaire, undergo blood sample collection for pro-inflammatory cytokines (TNF- α, IL-1β, and IL-6), event-related potential (ERP) detection in electroencephalogram (EEG), and fecal sample collection for microbiota analysis.

### Phase II of the study

Phase II of the study is a three-armed, parallel-group, single-blind, multicenter randomized control trial which aims to achieve the primary objective and secondary objectives of the study.

While for Phase II of the study which assess the differences in the primary and secondary outcomes between AUD patients allocated to the probiotic, ACT and placebo group across four timepoints (t_0_, t_1_, t_2_ and t_3_), the estimated sample size required was computed using the G*Power 3.1.9.7 calculator to estimate sample size needed for two-way repeated measure ANOVA: the α error was 0.05, the power was 0.8, the number of groups was 3, the number of measurements was 4 and the effect size was 0.15 [28]. Hence, the calculated sample size required for Phase II was a total of 120 subjects, which was 40 subjects per group (which included 30% of drop out rate).

#### Randomization

In Phase II of the study, a stratified permuted block randomization will be conducted where stratification according to age (18 to 30 years, 31 to 45 years old, and 46 to 55 years old) is performed and the experimental group subjects (n = 120) are randomized after 2 weeks of hospitalized detoxification to treat alcohol withdrawal syndrome into three groups, such as *Lactobacillus* sp. probiotic group, acceptance and commitment therapy (ACT) group, and placebo group in a 1:1:1 ratio. Randomization is according to computer generated randomization card which is concealed in a closed, opaque, sequentially numbered envelope. The randomization will be performed by a research assistant who is not involve in the study. Some amount of training will be provided to the research assistant on the procedures for randomization prior to that.

#### Interventions and data collection

**Fig 1:**
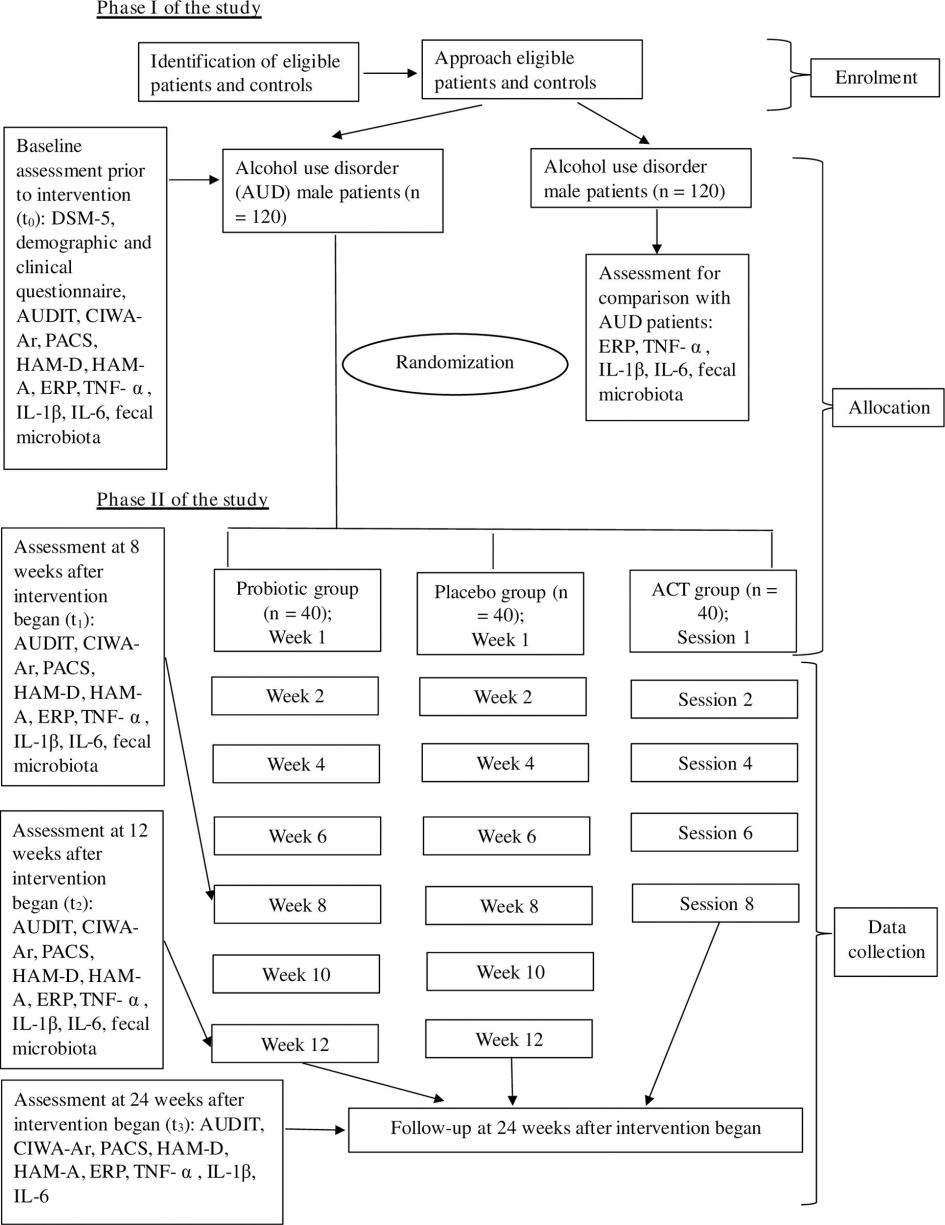
Flow chart of the study procedures and outcome measures. AUDIT = Alcohol Use Disorder Identification Test, CIWA-Ar = Clinical Institute Withdrawal Assessment for Alcohol-Revised, PACS = Penn Alcohol Craving Scale, HAM-D = Hamilton Depression Rating Scale, HAM-A = Hamilton Anxiety Rating Scale, ERP = event-related potential, TNF- α = tumor necrosis factor- α, IL-1β = interleukin-1β, IL-6 **=** interleukin-6, DSM-5 = Diagnostic and Statistical Manual for Mental Disorders 5^th^ Edition, t_0_ = pre-intervention assessment, t_1_ = 8 weeks after intervention began, t_2_ = 12 weeks after intervention began, t_3_ = 24 weeks after intervention began.

The subjects in the *Lactobacillus* sp. probiotic group will consume one sachet/day of *Lactobacillus* sp. for 12 weeks, where each sachet contains 9 log CFU of *Lactobacillus* sp. Participants will be given a product brochure before they start taking. The brochure contains product descriptions related to probiotics and placebo, how to take them, and how much to take. While the subjects assigned to the ACT group will undergo 8 sessions of ACT, one session per week where each session will last for about 50 minutes. Finally, the subjects in the placebo group will consume once sachet of placebo per day for 12 weeks, where each sachet only contains maltodextrin. Then, there are four time points of assessment, such as t_0_ (at baseline during Phase I of study, before the start of the interventions), t_1_ (8 weeks after the intervention begin), t_2_ (12 weeks after the intervention begin), and t_3_ (24 weeks after the intervention begin). The randomization and data collection of phase I and II of the study are illustrated in Fig 1.

1. *Probiotic treatment*. The probiotic product consists of a variety of strains of Lactobacillus spp. and maltodextrin which acts as a carrier. Each sachet of probiotic contains light-yellow powder which is stored in a place with surrounding temperature of below 25⁰C according to conditions recommended by the manufacturer. The subjects are required to keep the empty sachets after they consumed the probiotic and return to the research team for sachet calculation each time when they have a scheduled assessment.
2. *Placebo*. The placebo consists of only maltodextrin and flavoring agent which are in the same amount as the probiotic. Each sachet of placebo contains light-yellow powder and has a storage temperature similar to the probiotic which is at 25⁰C.

The probiotics and placebo will be administered to the subjects by one of the testers at a fixed time. The tester will be required to ensure that the temperature of the drinking water is below 37°C when the subject takes the probiotic or placebo.The tester is also required to collect the empty pouch promptly after the subject has taken the probiotic or placebo. Composition of the probiotic product and placebo are as follows in Table 1:

**Table 1:** Probiotics and placebo profiles.

Category	Probiotic Product	Placebo
Characteristics	Powder	Powder
Flavor	Grapefruit	Grapefruit
Strains	Lactobacillus Rosell^®^-52; Bifidobacterium longum Rosell^®^-175; Lactobacillus plantarum Rosell^®^-1012; Lactobacillus rhamnosus HA111	—
Strain Country of Origin Strain Manufacturer	Canada LALLEMAND	—
Specifications	1.5g: 10^10^CFU	1.5g: 0CFU
Directions	Pour directly into the mouth or mix with water below 37°C. Take 1 sachet daily 1 hour after lunch	
Product Manufacturer	Fan Le E-Commerce Co., Ltd., Shenzhen, China

*(3) Acceptance and commitment therapy (ACT)*. Initially, the ACT session began by aiming to establish good therapeutic rapport with the participant and explore in detail regarding the important personal information of the participant to formulate the case, such as external barriers, experiential avoidance, unworkable actions, fusion past and personal strength. The initial session began by coaching participants to acknowledge and accept their unpleasant thoughts and feelings which are associated with the urge to drink, and the symptoms related to AUD. Then, the participants will learn and practice on defusion which helps to decrease avoidance of unpleasant thoughts and feelings by acknowledging these problems and focus on pursuing values in life identified by the participants. In addition, mindfulness techniques will also be discussed and the participants will learn to apply mindfulness techniques to create awareness of the present and surrounding which are much enjoyable rather than to focus on effort employed to distract or avoid the unpleasant thoughts and feelings related to the urge to drink, and the symptoms related to AUD. The sessions use appropriate metaphors to enhance the understanding of the subjects and home assignments in the form of behavioral practices and exercises are provided at the end of the sessions in the form of pre-plan learning materials. The workbook provided to subjects as guidelines and for assignments are based on two books, which are “Get out of your mind and into your life” written by Hayes et al (2005) and “ACT made simple” written by Harris (2009) [37, 38]. The ACT module is summarized in S3 Appendix.

*Treatment fidelity for ACT*. The ACT sessions will be conducted by four therapists who are doctorate students in psychiatry (two in Xinxiang Medical University and two from Universiti Sains Malaysia) who have experience of conducting psychotherapy for at least two years. Initially, treatment manual will be provided to the therapists and they will undergo two full days of training workshop conducted by the principal investigator. Then, a psychiatrist and a clinical psychologist, both have formal training of ACT will measure the treatment integrity by examining 15% of the audio-visual recording of the therapists which was randomly selected by stratification according to therapists and the phase of the intervention (early, middle and late). The instrument which is use for assessing the treatment integrity is the Drexel University CT/ACT Therapist Adherence and Competence Rating Scale (DUACRS) [39]. Each assessor will evaluate half of the recordings. Then, the inter-rater reliability between the two treatment integrity assessors are evaluated to measure the treatment integrity of the therapists. Therapist who have difficulty or issues when conducting the ACT sessions will also seek advice from the principal investigator through discussion.

#### Blinding

This RCT follow single blinding whereby the researchers are blinded. The randomization will be performed by a research assistant, who had no contact with the patients and not involve in the research project. The research assistant will be trained to randomize subjects who first enrol in the study into the three groups (probiotic, ACT, and placebo groups) using computer system for randomization purpose. The allocation sequence of the participants will be blinded to all the researchers in the research team until the database has been completed and locked. Subject recruitment and data collection are performed by research assistant who is not involved in the study and not brief on the objectives of the study. The data analysis will be conducted by statisticians who is not involved in the study. Unblinding will only be unlocked once data collection and data analysis are completed. However, this study involved blinding of the researchers but not the blinding of the participants as ACT has no parallel control group. Unblinding of the researchers will be allowed if serious adverse effects related to the interventions in the study occurred.

#### Measures

The scheduled assessment of the study outcome measures are summarized in Table 2. Assessment of the study outcomes will be carried out by a research assistant who is not involved in the study and does not know the objectives of the study. The research assistant will received some amount of training to administered the measures before data collection begin. Assessment of the study outcomes will be performed in four time points, such as at t_0_ (baseline) which is prior to the interventions during Phase I of the study. Then in Phase II of the study, during t_1_ which is after 8 weeks the interventions began and t_2_ which is after 12 weeks of interventions began, the Chinese versions of the AUDIT, CIWA-Ar, PACS, as well as ERP detection in EEG, blood sample collection for pro-inflammatory cytokines (TNF- α, IL-1β, and IL-6), and fecal sample collection for microbiota analysis will be repeated. Finally during t_3_, which is 24 weeks after the interventions began, assessments are repeated with administration of instruments (AUDIT, CIWA-Ar, PACS, HAM-D, and HAM-A), ERP detection in EEG, and blood sample collection for pro-inflammatory cytokines (TNF- α, IL-1β, and IL-6). In order to enhance the adherence of the participants to the appointments for the serial assessments and intervention sessions, an appointment card is provided to the participants and emails or messages will be sent to participants 3 days prior to appointment date to remind them. If there is no reply from the participants, they will receive phone call to remind them of the appointment date. This clinical trial protocol and the scheduled assessment were prepared according to the Standard Protocol Items: Recommendations for Intervention Trials (SPIRIT) 2013 (The checklist of the SPIRIT is included in S4 Appendix).

**Table 2:** Scheduled assessments for the study’s outcome measures.

	Enrolment	Allocation	Post-allocation		
Time	*-t*_*1*_	*t*_*0*_ *(0 week)*	*t*_*1*_ *(8 weeks)*	*t*_*2*_ *(12 weeks)*	*t*_*3*_ *(24 weeks)*
**Enrolment:**					
**Eligibility screen**	X				
**Informed consent**	X				
**Allocation**		X			
**Interventions:**					
**(1) *Lactobacillus sp*. probiotic**			X	X	
**(2) Acceptance and commitment therapy (ACT)**			X		
**(3) Controls (treatment-as-usual)**			X	X	
**Assessments:**					
**(1) (a) Socio-demographic and clinical characteristics data**		X			
**(b) Alcohol use characteristics**		X	X	X	X
**(2) Primary outcomes:**					
**(a) Alcohol Use Disorder Identification Test (AUDIT)**		X	X	X	X
**(b) Clinical Institute Withdrawal Assessment for Alcohol-Revised (CIWA-Ar)**		X	X	X	X
**(c) Penn Alcohol Craving Scale (PACS)**		X	X	X	X
**(3) Secondary outcomes:**					
**(a) Hamilton Anxiety Rating Scale (HAM-A)**		X	X	X	X
**(b) Hamilton Depression Rating Scale (HAM-D)**		X	X	X	X
**(c) event-related potential (ERP) detection in electroencephalogram (EEG)**		X	X	X	X
**(d) Blood collection for pro-inflammatory cytokines (IL-6, IL-1β, TNF-α)**		X	X	X	X
**(e) Fecal collection for microbiota analysis**		X	X	X	

The outcome measures use in this RCT are as follow:

*(1) Socio-demographic*, *clinical and alcohol use characteristics questionnaire*. This questionnaire includes age, marital status, employment, education, monthly income, ethnicity, religion, history of medical illnesses, history of psychiatric illness, and history of medication intake, and duration of alcohol intake. In addition, the alcohol use characteristics include self-reported number of drinks per drinking day, the number of hazardous drinks on daily or weekly basis (for men, hazardous drinking is defined as more than 14 drinks per week or 4 drinks per drinking day; for women, hazardous drinking is defines as more than 7 drinks per week or 3 drinks per drinking days) [40], and frequency of binge drinking in the previous week (binge drinking is defined as more than 5 drinks on an occasion for men; and more than 4 drinks on an occasion for women) [41].

*Primary outcomes*.

(1) Alcohol Use Disorder Identification Test (AUDIT)- to assess alcohol use disorder:

This self-administered instrument consists of 10 items in three distinctive factors, whereby items 1 to 3 assess the amount and frequency of alcohol use, items 4 to 6 measure the severity of alcohol dependence and items 7 to 10 evaluate various problems related to alcohol use. In essence, a high score in items 1 to 3 and low scores in other items is indicative of harmful drinking, a high score in items 4 to 6 denotes greater severity of alcohol dependence, while a high score in items 7 to 10 shows problematic drinking. It has a cut-off score of ≥ 8 which denotes hazardous drinking [42]. The AUDIT was translated into Chinese and validated in the Chinese population of alcohol dependent users, where it exhibited an acceptable internal consistency with Cronbach’s α of 0.782 [43].

(2) Clinical Institute Withdrawal Assessment for Alcohol-Revised (CIWA-Ar)- to assess alcohol withdrawal:

This self-administered instrument is use to measure the severity of alcohol withdrawal symptoms. It consists of 10 items and each item is score in a Likert scale from 0 to 7. Higher total score indicates greater severity of alcohol withdrawal symptoms. A total score of < 10 depicts mild alcohol withdrawal, while a total score between 10 to 20 denotes moderately severe alcohol withdrawal, and a total score of > 20 indicates severe alcohol withdrawal which increase the risk of seizures and delirium tremens [44]. The CIWA-Ar was translated into Chinese and validated in the Chinese population of alcohol dependent users which exhibited a good internal consistency with Cronbach’s α of 0.83 [45].

(3) Penn Alcohol Craving Scale (PACS)- to assess alcohol craving:

This self-administered tool is made up of five items which measure several aspects of alcohol craving, such as the average degree of alcohol craving, its duration, intensity, frequency, and the difficulty experienced to cope with alcohol craving. A Likert scale is used to score each item wherein the score ranges from 0 which depicts none to 6 which denotes extremely severe. The subject is asked to score each item according to the situation in the past 1 week [46]. The PACS is translated into Chinese and validated in the Chinese population of alcohol dependent users which demonstrate an excellent internal consistency with Cronbach’s α of 0.97 [47].

*Secondary outcomes*.

(1) Hamilton Depression Rating Scale (HAM-D)- to evaluate depressive symptoms severity

The HAM-D is a researcher-administered instrument which measures depression in individuals before, during and after treatment. Although the HAM-D is made up of 21 items, only the first 17 items are scored. Each item is scored in a 5-point Likert scale or 3 -point Likert scale. Its total score ranges from 0 to 52. A total HAM-D score of 0 to 7 is indicative as normal, 8 to 16 depicts mild depression, 17 to 23 denotes moderate depression and a total score of 24 and above suggests severe depression [48]. The internal consistency of the Chinese version of the HAM-D was acceptable with a Cronbach’s α of 0.714 [49].

(2) Hamilton Anxiety Rating Scale (HAM-A)- to measure anxiety symptoms severity

The HAM-A is a clinician-administered questionnaire. It consists of 14 symptom-defined elements, and caters for both psychological and somatic symptoms, comprising anxious mood, tension, fears, insomnia; ‘intellectual’, depressed mood, somatic symptoms, sensory, cardiovascular, respiratory, gastrointestinal, genitourinary, autonomic, and observed behaviour at interview. Each item is scored on a basic numeric scoring of 0 (not present) to 4 (severe); with a total score range of 0–56, where <17 indicates mild severity, 18–24 mild to moderate severity and 25–30 moderate to severe. It has good psychometric properties [50]. The internal consistency of the Chinese version of the HAM-A was excellent with a Cronbach’s α 0.93 [51].

**Fig 2:**
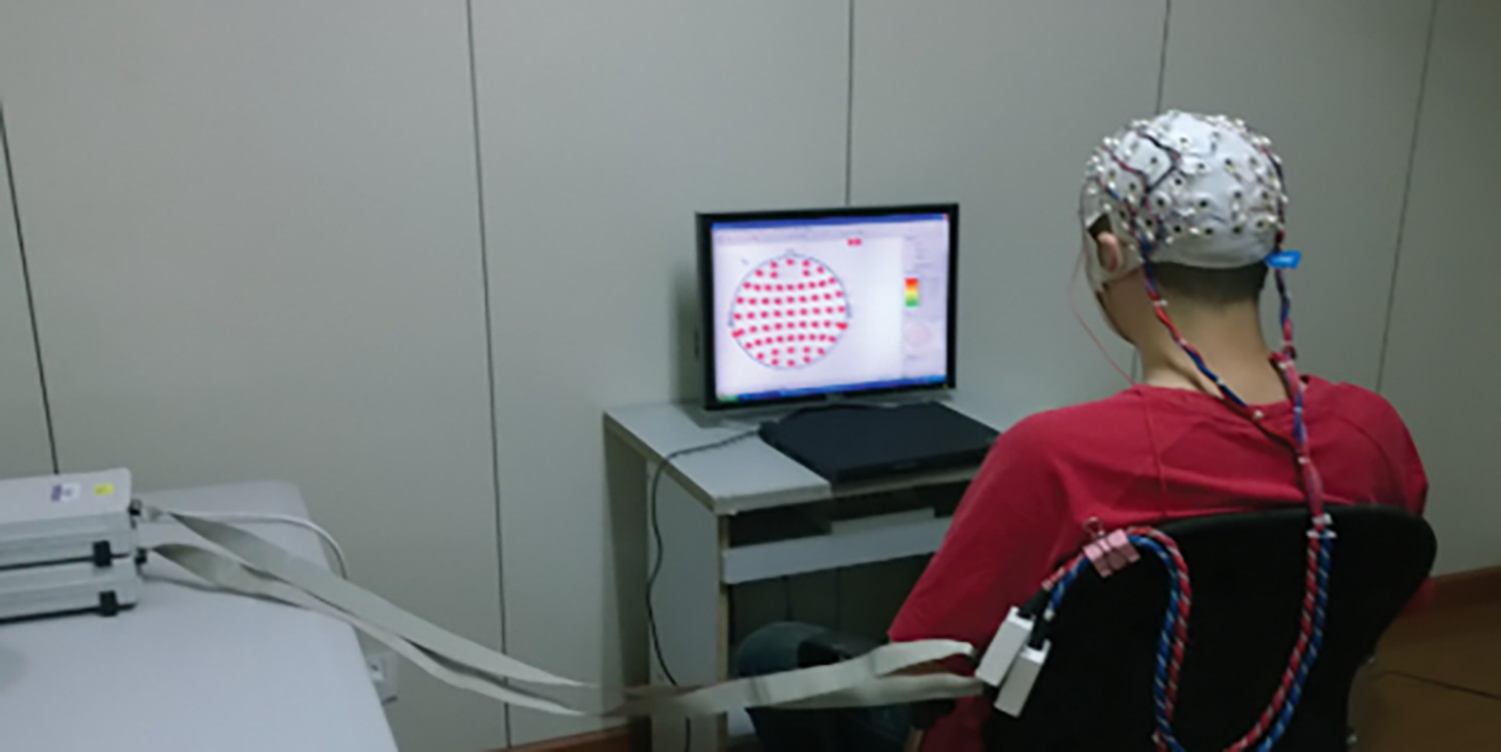
Administration of visual stimuli to participant during event-related potential (ERP) detection in electroencephalogram (EEG).

(3) Event-related potential (ERP) detection in electroencephalogram (EEG)
  (a) Experimental paradigm:

Visual stimuli will be presented by E-Prime 2.0 software. The paradigm consists of three types of images such as i) images related to alcohol cues (common drinking environments, habitual drinking products, etc.), ii) neutral images unrelated to alcohol cues, and iii) task-related images requiring key operation. We will collect EEG while patients watch the stimulation paradigm (Fig 2) [52].

**Fig 3:**
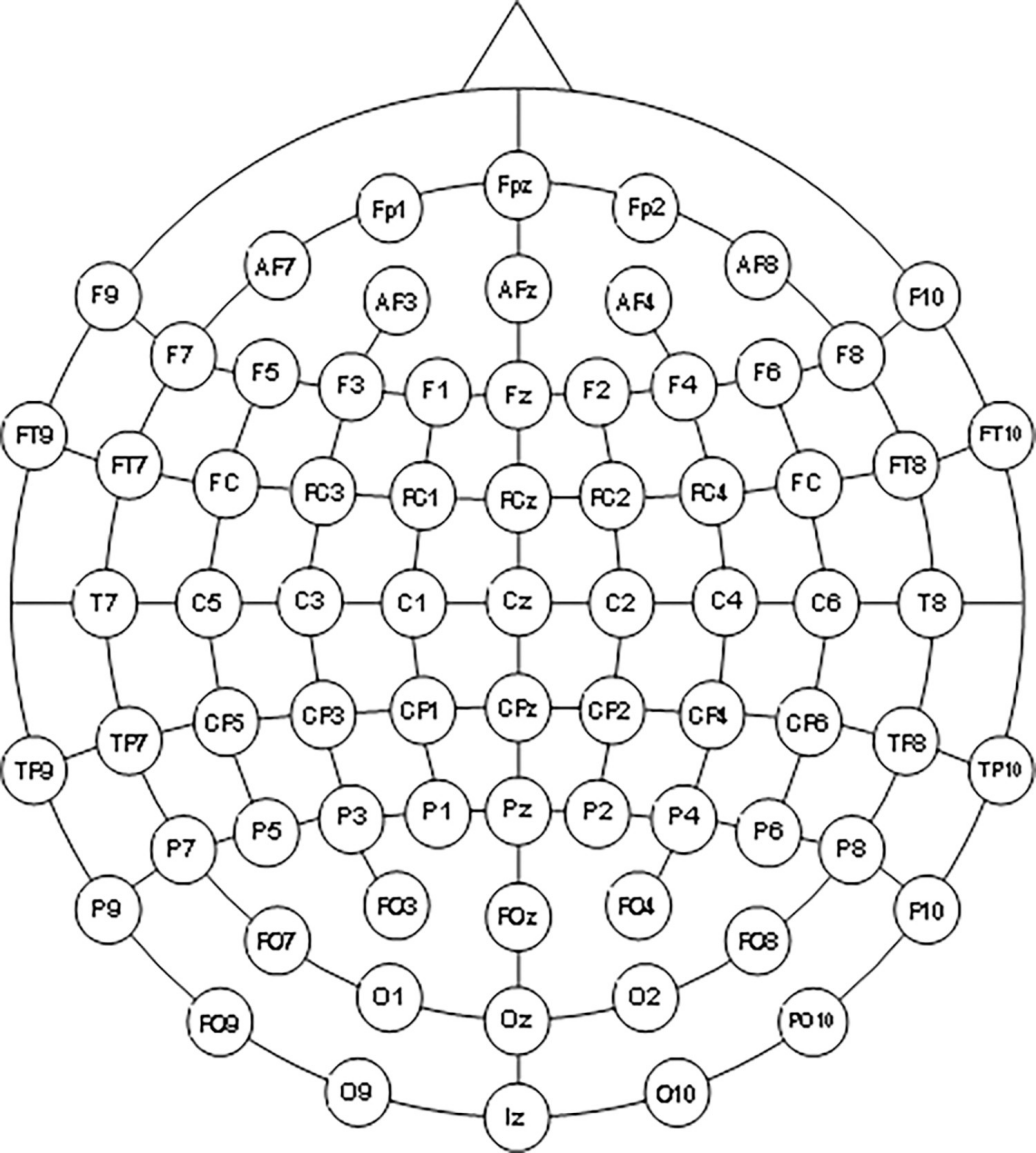
The 64 standard scalp positions recorded during event-related potential (ERP) detection in electroencephalogram (EEG).

(b) EEG signal acquisition:

We will use the Brain Amp MR-32 instrument to collect signals, and record data through the Brian Vision Recorder software. 64 standard scalp positions will be recorded according to the 10–10 standard lead system (Fig 3). The sampling rate is 1000 Hz, and the impedance between electrode and skin should be less than 5 kΩ. [52].

(c) ERP analysis:

Data will be analyzed using Brian Vision Analyzer 2.1 software. The process includes ① re-reference, ② filtering, ③ removal of ocular artifacts, ④ artifact removal, ⑤ segmentation, ⑥ baseline correction, and ⑦ peak detection.

(4) Serum pro-inflammatory cytokines analysis:

Blood sample of about 5 ml will be collected from each participants during each assessment. Serum pro-inflammatory cytokines (TNF- α, IL-1β, and IL-6) levels among the participants were assessed using enzyme-linked immunoabsorbent assay (ELISA). The serum sample is diluted with sufficient buffer from the ELISA kit according to the instructions of the manufacturer. The concentration of the sample is read against a standard curve and is expressed as concentration per unit of the sample. While the gene expression of the pro-inflammatory cytokines is evaluated with real time polymerase chain reaction [52].

**Fig 4:**
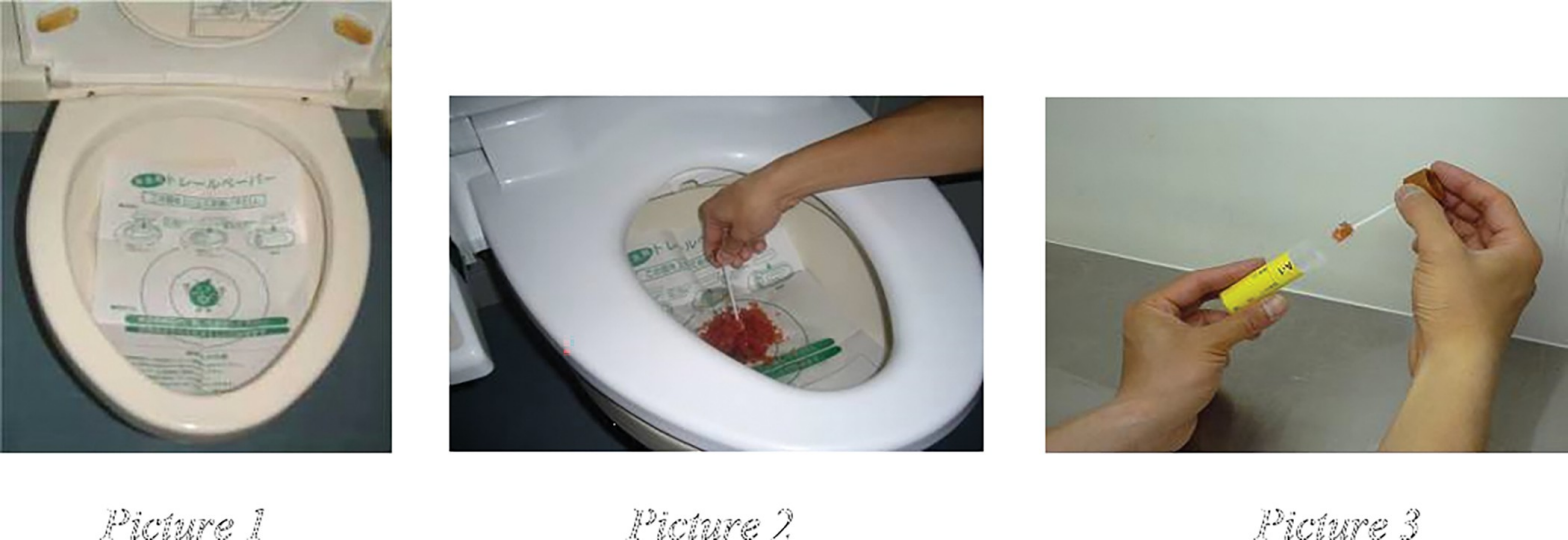
The procedures in which the fecal sample is collected illustrated in Picture 1, 2, and 3.

(5) Fecal microbiota analysis:

Gut microbiota content is evaluated by fecal microbiota analysis among the participants. At about two weeks before fecal sample collection, the participants are brief on fecal sample collection. In addition, the participants will also receive picture instruction leaflet on fecal sample collection to guide them. They will be provided with fecal collection tube (which consists of RNAlater™ solution and 4 glass beads in the tube) and rice paper. As for the steps of fecal sample collection, initially, the rice paper is floated on the water surface of the lavatory bowl (refer Picture 1 of Fig 4). Then, two spatula portions of the feces which is defecated on the rice paper are collected using the spatula which is attached to the tube (refer to Picture 2 of Fig 4). The participant will have to ensure that the fecal sample is not contaminated with mineral oil, urine, barium or water. Finally, the spatula is placed into the fecal collection tube and capped tightly (refer to Picture 3 of Fig 4). The tube is then placed in a zip-lock bag, sealed and frozen at -80⁰C or in liquid nitrogen. The fecal sample collection should be completed within one week after the participants received the fecal collection tube and rice paper. Hence, the fecal sample should be collected no later than 1 week after participants received the fecal collection tube. After the participants have collected the fecal sample, the research team will arrange courier team to take the sample from the subjects and to be brought back to the lab within few hours [52].

The 16s rRNA analysis method will be used for the analysis of intestinal microbiota.The PCR reaction system was configured with qualified genomic samples and corresponding fusion primers, and the corresponding PCR parameters were set to perform PCR amplification, and the PCR amplified products were purified to complete the library construction. The fragment range and concentration of the library were detected by Agilent 2100 Bioanalyzer. The qualified libraries were sequenced on the DNBSEQ platform. The downstream data were filtered, and the remaining high-quality Clean data could be used for later analysis. The reads were spliced into Tags by the Overlap relationship between the reads.The Tags were clustered into OTUs at a given similarity, and then the OTUs were compared with the database to perform species annotation. Based on the OTUs and species annotation results, alpha diversity analysis, beta diversity analysis, and species difference analysis will be performed.

*Other measures*. The participants’ compliance to the ACT intervention will be documented in electronic diaries which they are expected to fill in their electronic dairies to report on their home assignments and practices given during each session of ACT. Any missed sessions and reasons for drop-out or loss to follow up (such as uninterested to continue intervention, personal isssues or problems, transportation problem, and poor health) will be documented.

All participants are also monitored for consumption of food or diet which are classified as probiotic and prebiotic during the duration which they participate in the study, such as yogurt, sauerkraut, whole grains, bananas, greens, onions, garlic, soybeans and artichokes; whereby any intake of these food should be reported in their electronic diaries.

### Data analysis

All data analysis will be performed using SPSS software version 26.0 (SPSS Inc, Chicago, IL, USA). The descriptive statistics for sociodemographic and clinical characteristics, the total CIWA-Ar, AUDIT, PACS, HAM-A and HAM-D scores, the serum levels of IL-1β, IL-6 and TNF-α; and the ERP on EEG of the participants will be reported. Categorical data will be reported as percentage and frequency. While continuous data will be recorded as mean and standard deviation if the data are normally distributed, otherwise continuous data will be presented as median and interquartile range if the data are non-normally distributed. In Phase I of the study, the differences in the serum level of IL-1β, IL-6 and TNF-α between experimental group and healthy controls will be evaluated using independent t-test if normally distributed or using Mann-Whitney U test if non-normally distributed.

In phase II of the study, the mean difference in the primary outcomes (the CIWA-Ar, PACS, AUDIT scores, and alcohol use charactetistics, such as self-reported number of drinks per drinking day, the number of hazardous drinking on daily or weekly basis, and the frequency of binge drinking in the previous week) for the three randomized groups (ACT, probiotic, and palcebo groups) at each specific time point (pre-intervention [t_0_], 8 weeks after intervention began [t_1_], 12 weeks after intervention began [t_2_], and 24 weeks after starting intervention [t_3_]) will be evaluated using one way analysis of variance (ANOVA) and followed by false discovery rate adjustment. If the primary outcomes are not normally distributed, then Kruskal-Wallis test is performed and followed by Dunn’s multiple comparison test.

Regarding the main pool analysis, mixed ANOVA will be utilized to determine the interaction between the three randomized groups (ACT, probiotic, and placebo groups) and the four time points (t_0_, t_1_, t_2_, and t_3_) on the primary outcomes (such as the CIWA-Ar, PACS, and AUDIT scores; whereby interaction = intervention × time; where time is a within-subject variable and intervention effect is a between-subject variable). The main effects of intervention in the groups and time points will be reported as estimated marginal mean and standard error of mean. If the primary outcomes are not normally distributed, then a non-parametric mixed-effect ANOVA is performed with the R package nparLD statistical software [53]. The primary analysis will abide by the intention-to-treat analysis. The data analysis for the secondary outcomes (severity of anxiety and depressive symptoms, pro-inflammatory cytokines, EEG characteristics, and fecal microbiota) will be performed similar to the primary outcomes’ analysis. Statistical significance is set at p < 0.05 and will be two-tailed.

In subgroup analysis, we use linear mixed-effect model to assess the association between the fecal microbiota and the ERP changes in EEG across the three timepoints (t_0_ to t_2_) while controlling for confounding factors such as self-reported number of drinks per drinking day, the number of hazardous drinks on daily or weekly basis, and frequency of binge drinking in the previous week in each of the three groups of intervention. Multiple comparison correction are achieved by running the Bonferroni correction. If the fecal microbiota count is not normally distributed, then logarithmic transformation of data is warranted before the linear mixed-effect model is performed.

In the handling of missing data, if only 5% to the total data collected is missing, then this missing data will be ignored. If the missing data is around 5% to 40% and if it is assumed to be randomly missing, then multiple imputation with restricted maximum likelihood estimation will be utilized (using Stata 15). However, if the missing data is more than 40% of the total data collected or it is assumed not randomly missing or completely randomly missing, then the missing data will be ignored and data analysis with the available data will proceed. The missing data issue will be reported as limitation of the study during publication or reporting of the research findings [54].

### Data availability

Anonymous individual data will be made available after publication of the study findings. The data will be uploaded into the Figshare data repository after publication of the study findings.

## Discussion

While alcohol abstinence is desirable as the ultimate outcome of AUD treatment, only 16% of patients with AUD are able to achieve abstinence [55]. Existing studies on the treatment of AD found that the effect of drug therapy on alcohol craving is very limited. The US Food and Drug Administration (FDA) has approved three drugs for abstinence: disulfiram, naltrexone and acamprosate. As a type of alcohol antagonist, disulfiram causes patients to experience strong physical discomfort after ingesting a small amount of alcohol, which may place patient at risk of the disulfiram-ethanol interaction reaction, at high risk of non-compliance and cannot effectively reduce craving, so it is basically not a preferred treatment for AUD unless supervised treatment with disulfiram can be carried out [55]. Similarly, the efficacy of naltrexone and acamprosate are also suspected and inconclusive for preventing relapse in AUD [55]. In view of the high relapse rate and rising prevalence of AUD, it is of great significance to investigate new treatment methods that can effectively intervene in alcohol craving, which should be easy to be applied in clinical practice.

One of the potential novel treatment for AUD is probiotic. On the gut-brain axis, intake of prebiotics and probiotics was beneficial for neurochemical changes in rats, including increased hippocampal expression of brain-derived neurotrophic factor and glutamate receptors [56], which are involved in the regulation of many behaviors, such as anxiety, depression, cognitive performance, and addiction [57]. Recent research suggests that gut bacteria may influence brain function and behavior. As demonstrated by Temko et al. (2017), alcohol dependence, other substance use disorders, and eating disorders are all associated with changes in neurobiological pathways in specific regions of the brain involved in reward processing, and changes in gut microbiota may alter biological pathways by reducing systemic inflammation [20]. However, Leclercq et al. (2019) showed in their review that the effects of intestinal microbiota on the neurobiological processes of patients with substance use disorder still need to be further studied, but in essence, probiotics or prebiotics are a promising treatment method for alcohol dependence and substance dependence [16].

Potentially, the use of ACT for treatment of SUD has reported some successes [58]. In an uncontrolled pilot study of 43 US veterans with posttraumatic stress disorder and alcohol use, 12 sessions of ACT as compared to treatment-as-usual showed reduction in the number of drinks and heavy drinking days at the end of the ACT intervention. However, this study was limited by small sample size (n = 43), lack of a control group, and high attrition rate at 33% [59]. While in a case report of a 29-year-old college student with AUD, ACT intervention reported to reduce the number of drinking episodes and severe drinking at the end of the intervention. However, the above is a case report where its findings could not be generalized to represent the AUD population [60]. Hence, it would be interesting to investigate the efficacy of ACT for treatment of AUD in a randomized controlled trial to fill the research gap.

This study compares the efficacy of probiotic and ACT in alleviating the severity of alcohol craving and AUD among patients who had undergo two weeks of in-patient detoxification. In addition, this study also compares the efficacy of probiotic and ACT in mitigating the severity of comorbid depression and anxiety symptoms; decreasing serum level of pro-inflammatory cytokines, such as IL-1β, IL-6, and TNF-α; changing the event-related potential in EEG and restoring microbiota in the gut of AUD patients. Data on the efficacy of probiotic and ACT to reduce alcohol craving and severity of AUD is lacking.

However, there are a few limitations of this study to take note of. First, this study will only recruit Chinese patients with Han ethnicity in China and Malaysian Chinese patients with Han ethnicity. Hence, the study sample may not be generalized to represent the AUD population in China and Malaysia. Second, this study will recruit only male patients and again the study findings may not be generalized to the AUD population in China and Malaysia. Nevertheless, there is a wide gender differences in the prevalence of AUD in China and Malaysia, whereby male predisposition for AUD are evidenced in both countries [61, 62].

Despite the limitations of the study, the study findings will provide significant clinical implications. If efficacy of both novel modes of treatment (probiotic and ACT) for AUD are documented in this study, it will provide treating clinicians with more treatment options for AUD patients. These two modes of treatment may be incorporated into the treatment regimen for AUD to prevent relapse and maintain patients on abstinence from alcohol use. Moreover, the methodology of this study can be replicated for future studies to investigate the efficacy of probiotic and ACT in mitigating craving and severity of other types of substance use disorder.

## Supporting information

S1 Appendix The research protocol approved by the Human Research Ethics Committee of Xinxiang Medical University and the Human Research Ethics Committee of Universiti Sains Malaysia. (DOC)

S2 Appendix Participant information sheet and consent form use in this study (English version). (DOC)

S3 Appendix Summary of the ACT module in this study. (DOCX)

S4 Appendix Checklist of the Standard Protocol Items: Recommendations for Intervention Trials (SPIRIT) 2013. (DOC)
