# Supplementary material for: Research protocol of the efficacy of probiotics for the treatment of alcohol use disorder among adult males: A comparison with placebo and acceptance and commitment therapy in a randomized controlled trial
Source: PLoS One. 2023 Dec 5;18(12):e0294768. doi: 10.1371/journal.pone.0294768 (PMC10697511; doi:10.1371/journal.pone.0294768)
Supplement: S1 Appendix — (DOC) [file pone.0294768.s001.doc]

**Additional file 1. The research protocol approved by the Human Research Ethics Committee of Xinxiang Medical University and the Human Research Ethics Committee of Universiti Sains Malaysia**

**Research title: The Efficacy of Probiotics for the Treatment of Alcohol Dependence (AD) among Adult Males: A comparison with Placebo and Acceptance and Commitment Therapy (ACT)**

**Principal investigator (MMC No. if applicable): Zhang Bingyu and Dr. Mohammad Farris Iman Leong Bin Abdullah (MMC: 43103)**

**Co-researchers: (MMC No. if applicable): Dr. Nurul Izzah Shari (UTM)**

**Introduction**

Alcohol is a common and easily accessible addictive substance. There are a large number of drinkers worldwide, and the impact of alcohol-related problems cannot be ignored. According to the latest Global Status Report on Alcohol and Health 2018 released by the World Health Organization (WHO) [1], there are about 2.3 billion drinkers in the world at present. In 2016 alone, about 3 million deaths could be attributed to the harmful use of alcohol, accounting for 5.3% of the total number of deaths, while drinking was also responsible for 132.6 million disability-adjusted life years (DALYs). Alcohol consumption is related to more than 60 kinds of diseases and injuries, mainly involving cancer, cardiovascular diseases, digestive diseases, traffic accidents and intentional injuries, etc. It is the third pathogenic risk factor after hypertension and smoking [2]. With the development of economy, the production and consumption of alcoholic beverages increased rapidly. According to the report of WHO [1], the per capita alcohol consumption in China soared from 4.1 liters in 2005 to 7.2 liters in 2016, while the lifelong abstinence rate decreased from 50.9% in 2005 to 42.1% in 2016. The prevalence of alcohol use disorder (AUD) in China is also increasing year by year. At present, the prevalence of AUD is 6.9% in males and 0.2% in females [3]. Alcohol-related problems have become one of the important public health problems in China, and the situation is quite serious.

**Problem statement & Study rationale**

AD is a chronic and recurrent mental disorder. It will affect the brain's ability to control emotions, decisions and behaviors, resulting in the decline of work and learning abilities, impaired physical and mental health, and even legal problems. AD poses a serious threat to individual health, family well-being and social stability. Therefore, how to effectively treat the disease and prevent relapse is of great significance. So far, many studies on alcohol addiction have not been able to fully elucidate its pathogenesis, which is generally considered to be caused by neurobiological, psychological and sociological factors [4]. Previous studies have suggested that the most important neuroadaptive changes in the progression from occasional drinking to addiction may be down-regulation of the dopamine (DA) and gamma aminobutyric acid (GABA) systems, as well as permanent up-regulation of the glutamate (Glu) system, in addition to disruption of the brain's stress system: Dysregulation of corticotropin releasing hormone (CRH) and serotonin (5-HT) [5]. Other studies have found that alcohol sensitivity is closely related to addiction, and prolonged alcohol intake can reduce the body's response to alcohol, which is one of the risk factors leading to AD [6].

In addition, studies on the mechanism of addiction tend to be based on the theory of "reward system", which mainly involves the mesolimbic dopamine system [7] and endogenous opioid system [8]. Studies on psychological and sociological factors consider that alcohol addiction is related to psychological reinforcement, personality traits, alcohol accessibility, family environment, peer influence and social cultural background, etc. [9-11].

The core symptoms of AD mainly include two aspects: one is physical dependence based on increased alcohol tolerance and withdrawal reaction, and the other is psychological dependence based on alcohol craving [4]. Currently, clinical treatment for AD is focused on reducing physical dependence. Benzodiazepines (BZD) are usually given to alleviate withdrawal symptoms, low-dose antipsychotics to relieve psychotic symptoms, and adequate vitamin B supplementation to prevent Wernicke encephalopathy. However, most patients still have a strong craving for alcohol during abstinence, even after receiving standardized in-patient treatment. This psychological dependence on alcohol is hard to resist, so it is extremely difficult to maintain long-term abstinence, and AD is prone to relapse. Therefore, psychological dependence is the key factor leading to alcoholics drinking again after abstinence, and the monitoring and treatment of alcohol craving is an important link to improve the therapeutic effect of AD.

Existing studies on the treatment of AD have found that the effect of drug therapy on alcohol craving is very limited. The US Food and Drug Administration (FDA) has approved three drugs for abstinence: disulfiram, naltrexone and acamprosate. As a kind of alcohol sensitizer, disulfiram causes patients to appear strong physical discomfort after ingesting a small amount of alcohol, which has a high risk and cannot effectively reduce craving, so it is basically no longer used clinically.

In view of the high relapse rate and rising prevalence of AD, it is of great significance to find new methods that can effectively intervene in alcohol craving, which should be easy to be popularized and applied in clinical practice. New therapies should integrate biological, psychosocial and environmental factors in order to improve the abstinence rate and reduce the relapse rate. It will provide a breakthrough in the treatment for AD and even the field of addiction medicine.

The treatment and detection methods adopted in this study include probiotics treatment, intestinal microflora detection, ERP detection, and multi-parameter physiological index detection. All of the above are emerging technologies, tools and research hotspots in recent years, and all of them have the advantages of safety, and non-invasive.

Probiotics therapy is a simple, safe and low-cost treatment method, which can restore intestinal flora homeostasis, repair intestinal barrier, improve nutritional status and other physical conditions of patients to a certain extent. It is expected to regulate patients' mood, reduce patients' craving for alcohol and reduce the recurrence rate through the gut-brain axis. Intestinal flora detection can also monitor the changes of patients' flora, evaluate the therapeutic effect of probiotics, and hopefully analyze the main flora affecting the enteric-brain axis in AD patients, providing a theoretical basis for further research.

ERP detection has excellent time resolution and can objectively evaluate the psychological activities of patients. The application of this technology has the advantages of high efficiency, economy, high sensitivity and non-invasive to subjects. In addition, combined with EEG traceability analysis, it can study the characteristics of alcohol craving. The combination of these emerging therapeutic methods and detection methods in this study will help break through the bottleneck of AD treatment and prevention at the present stage, optimize the treatment plan, improve the quality of life of AD patients, reduce the social burden, and produce obvious social and economic benefits.

**Research Question(s)**

1. Does probiotic or ACT result in significant improvement in biological markers, and reduce the degree of AD symptoms severity among patients diagnosed with alcohol dependence after 8 weeks of intervention?

2. Does probiotic or ACT result in significant improvement in rate of alcohol repetition and alcohol withdrawal symptoms and degree of craving?

3. Whether ACT treatment or probiotics can cause EEG changes in alcohol dependent patients?

**Objective**

**Primary objective:**

To determine the differences in the severity of alcohol dependence, alcohol withdrawal and craving among patients on Lactobacillus sp., placebo and ACT, at 4 timelines (baseline, 8 weeks after starting intervention, 12 weeks after starting intervention, and 24 weeks after starting intervention which is 12 weeks post termination of intervention).

**Secondary objectives:**

(1) To determine the differences in the serum levels of pro-inflammatory cytokines among patients on Lactobacillus sp., placebo and ACT, at 4 timelines (baseline, 8 weeks after starting intervention, 12 weeks after starting intervention, and 24 weeks after starting intervention which is 12 weeks post termination of intervention).

(2) To assess the differences in severity of depression and anxiety symptoms among patients on Lactobacillus sp., placebo and ACT via use of questionnaire, at 4 timelines (baseline, 8 weeks after starting intervention, 12 weeks after starting intervention, and 24 weeks after starting intervention which is 12 weeks post termination of intervention).

(3) To investigate the changes of EEG characteristics in patients with AD after the above related treatments, at 3 timelines (baseline, 2 weeks after starting intervention, 8 weeks after starting intervention).

(4) To assess the differences in gut microbiota profiles of patients on Lactobacillus sp., placebo and ACT via the use of fecal samples, at 3 timelines (baseline, 2 weeks after starting intervention, and 8 weeks after starting intervention).

**Literature review**

**2.1 Relationship between intestinal flora and alcohol dependence**

Over the past decade, the link between the gut, and specifically the gut microbiome, and alcohol dependence has come to the attention of researchers. In individuals with alcohol addiction, drinking can disrupt intestinal barrier function, also known as intestinal leakage[12-13]. The intestinal barrier consists of intestinal cells, goblet cells, and antibacterial substances that influence the intestinal microbiota within the mucus layer, as well as many immune cells in the lamina propria[14]. The mechanism by which alcohol or its metabolites cause intestinal leakage is unknown, and may be related to intestinal disorders, immune system activation, and inflammation. Dysbiosis of intestinal flora refers to changes in intestinal flora, which is characterized by changes in intestinal bacterial groups, such as decreased levels of anti-inflammatory bacteria and increased abundance of Proteus[15-16]. Animal studies have shown that improved intestinal barrier integrity can ameliorate alcohol-induced liver injury[17-19]. This suggests that treatment modulated by the gut microbiome, such as probiotics, may be beneficial for patients with AD. Persistent alcohol abuse can change fecal pH, promote excessive growth of pathogens, and alter the function of intestinal microorganisms by altering the secretion of specific metabolites involved in intestinal barrier dysfunction [20-21]. Elevated levels of plasma cytokines such as TNF α , interleukin and CRP in PATIENTS with AD suggest chronic, low-grade, systemic inflammation[22]. In the past decade, several studies have suggested a link between systemic inflammation and psychiatric disorders such as alcohol addiction, depression, and autism, and the gut microbiome could be a good target for research. One possible mechanism of systemic inflammation and alcohol addiction is that intestinal bacterial products activate peripheral blood monocytes and induce cytokines to enter the blood, leading to alcohol addiction and other psychiatric disorders, including major depressive disorder, bipolar disorder and anxiety disorder[23]. The severity of alcohol addiction is related to the intensity of patients' cravings, cognitive dysfunction, anxiety and depressive symptoms.

Thus, systemic inflammation may play an important role in the development of alcohol addiction, but causality remains unclear. However, alcohol cravings and negative emotions such as anxiety and depression were highly associated with alcohol-seeking behavior and relapse, suggesting that reducing systemic inflammation may improve mental health and prevent relapse. Probiotics are healthy microbes, and alcohol dependent patients had fewer bifidobacteria and lactobacillus in their gut than the normal population. Oral supplementation of good bacteria can help restore gut flora and improve alcohol-related illness. In ethanol-fed rats, lactobacillus GG supplementation can reduce endotoxemia and alcohol-induced liver injury[24]. In alcohol-dependent patients, supplementation of Bifidobacterim bifidum and Lactobacillus plantarum 8PA3 during alcohol detoxification had a greater effect on the reduction of liver enzymes than dietary restriction alone[25]. In patients with alcoholic cirrhosis, administration of lactobacillus casei Shirota improved neutrophil phagocytosis compared with conventional treatment[26]. In addition, in patients with alcohol use disorders, administration of VSL#3(a mixture of eight different strains), Compared with traditional treatment, plasma levels of TNF α , IL-6 and IL-10 were significantly reduced[27]. These data suggest that probiotics have beneficial effects on the enteric-liver axis in alcohol-dependent patients. Several clinical trials have demonstrated beneficial effects of probiotics on psychological symptoms and brain activity[28]. On the gut-brain axis, intake of prebiotics and probiotics was beneficial for neurochemical changes in rats, including increased hippocampal expression of brain-derived neurotrophic factor and glutamate receptors[29], which are involved in the regulation of many behaviors, such as anxiety, depression, cognitive performance, and addiction[30]. Recent research suggests that gut bacteria may influence brain function and behavior. As demonstrated by Temko et al., alcohol dependence, other substance use disorders and eating disorders are all associated with changes in neurobiological pathways in specific regions of the brain involved in "reward" processing[31], and changes in gut flora may alter biological pathways by reducing systemic inflammation. However, Sophie Leclercq et al. showed in their review that the effects of intestinal flora on the neurobiological processes of patients with substance use disorder still need to be further studied, and probiotics or prebiotics are a promising treatment method for alcohol dependence and substance dependence.

**2.2 Acceptance and commitment therapy (ACT)**

ACT is a third generation therapy, which is new from cognitive behavioral therapy (CBT), and it is approach to produce psychological flexibility. Psychological flexibility is defined as the ability to contact the present moment more fully as a conscious human being and to change or persist in behavior when doing so serves valued ends [32]. Unlike CBT, which aims to change unhelpful thoughts and feelings. ACT was designed to increase adaptive coping through acceptance, cognitive defusion, mindfulness, and perspective-taking exercises while supporting clients in aligning behavior with their personal values [33-34]. ACT facilitates development and maintenance of health behavioral improvements by targeting internal barriers [35]. ACT can be used to treat depression and anxiety by teaching patients the right way to deal with negative emotions and relieve psychological symptoms.The alcohol-seeking behavior of AD patients is often associated with anxiety and depression. It may be possible to reduce the frequency of alcohol-seeking behavior by alleviating bad mood.Therefore, the use of ACT in the treatment of AD patients is a worthwhile approach.

**2.3 Research Progress of Event-related Potential Technology**

Event-related potential (ERP) is a special evoked EEG. When the body receives multiple or diverse events with psychological significance, there will be a "time-locked" relationship between the EEG and the stimulation, which can reflect the neural electrophysiological phenomenon of the brain in the process of cognitive processing. ERP is an "online window" to observe the mental activities of human brain, which can make objective evaluation of advanced mental activities[36]. In the past, many scholars have used ERP technology to carry out studies on substance addiction, especially in the composition of ERP-P300. P300 is associated with a variety of cognitive processes and is an indicator of a variety of neurocognitive processes, including attention and working memory. Some studies suggest that abnormal ERP amplitude may be a disease marker of substance dependence and a potential neurobiological endophenotype[37]. Bartholow et al. used an experimental paradigm containing images with alcohol cues to detect P300, and found that the P300 response of subjects with low sensitivity to alcohol was significantly enhanced, while that of subjects with high sensitivity to alcohol was not.

Moreover, such enhanced P300 response was alcohol-specific and would not be extended to other motivational related stimuli. Alcohol cue-induced P300 may be a sensitive endophenotype for the risk of alcohol addiction[38]. Petit et al. 's study showed that alcoholics had enhanced ERP responsiveness to alcohol-related cues, especially male patients[39]. Meta-analysis on drug dependence indicated that in the detection of attention preference and craving in drug cue images, the increase of cue-induced P300 amplitude was correlated with the increase of subjects' craving degree[40]. The previous study of our research team[41] found that there were positive results in EEG analysis of AD patients in the craving state induced by static pictures of alcohol cues, that is, AD patients presented lower N200 amplitude and higher P300 amplitude under the stimulation of alcohol cues, which were positively correlated with the subjective craving scale score. It is possible to objectively assess the psychological dependence of AD patients by using alcohol cue response to induce the craving for alcohol and monitoring the task-state EEG (ERP) of the subjects at the same time.

In conclusion, drinking alcohol can change the intestinal barrier and intestinal flora of AD patients. Changes in intestinal flora have a certain degree of influence on AD patients, but the specific mechanism remains to be further studied and clarified. Regulation of gut microbiota through the use of probiotics or prebiotics is a promising and safe treatment for AD. Prospective randomized controlled clinical trials are still needed to evaluate the effects of probiotics and/or prebiotics on AD, especially on the different behavioral manifestations of AD. Such as studying the effects of probiotics and/or prebiotics on depression, anxiety, stress response, cognition, impulsivity, and alcohol-seeking behavior in patients with AD. In addition, ERP is an economical, non-invasive and objective method to reflect the psychological dependence of AD patients. ERP testing in conjunction with studies of probiotics and/or probiotics in the treatment of alcohol-dependent patients may help to better evaluate and reveal the specific impact of intestinal flora on patients with AD.

**Conceptual framework**


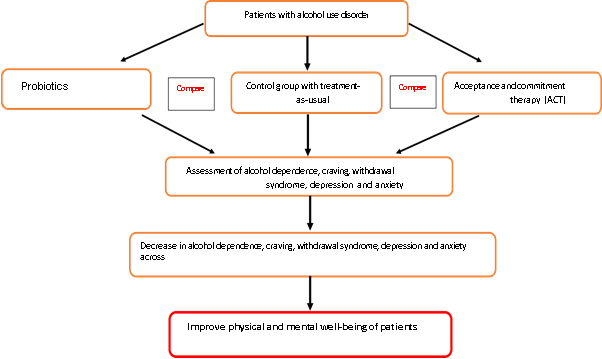


**Research design**

A double-blind randomized controlled trial design has been chosen.

**Study area**

The study will be completed in the Second Affiliated Hospital of Xinxiang Medical University, Xinxiang, Henan, China.

**Study population**

In this study, patients with alcohol dependence who met the diagnostic criteria of "alcohol use disorder" in DSM-5 were selected as the experimental group. Advertisement with posters will be placed on display in the 2^nd^ Affiliated Hospital of XXMU and the Department of Psychiatry, XXMU to help in the recruitment of subjects. Healthy people who met WHO healthy drinking standards or did not drink were used as the healthy control group. The experimental group was divided into probiotics + routine treatment group and placebo + routine treatment group and ACT + routine treatment group.

**Subject criteria**

A) The AD subjects

a) Inclusion criteria:

-Hospitalized patients diagnosed with untreated alcohol use disorder (confirmed by the diagnostic criteria of DSM-5).

-Male, age 18 to 55 years old, Han nationality, junior high school education or above,

right-handed.

-Those with normal eyesight, including corrected vision(screened clinically).

b) Exclusion criteria:

-The patient suffer from other mental illnesses (screen by DSM-V criteria).

-The patient has allergy reaction to medications use for conventional treatment of alcohol use disorder.

-The patient had a history of organic brain disease, a pacemaker, gastrointestinal surgery, or serious health problems.

-The patient had a history of seizures.

-The patient is complicated with severe physical disease.

-Patient has other drug dependence (except for nicotine dependence).

-The patient took drugs affecting intestinal flora 30 days before and during admission.

-The patient has participated in any other alcohol-related studies or trials within the past

30 days.

-Patients had use any prescription or over-the-counter drugs in the past 30 days that may

affect mood or alcohol cravings.

B）Healthy control group

a) Inclusion criteria:

-Male, age 18 to 55 years old, Han nationality, junior high school education or above,

right-handed.

-No history of psychoactive substance abuse, except tobacco (screen with urine dipstick test).

-Those with normal eyesight, including corrected vision (screen clinically).

-No alcoholic beverages in the last 2 weeks.

-According to the WHO healthy alcohol consumption standard, the average intake of

pure alcohol per week is less than 210 grams, or do not drink at all (screen clinically).

b) Exclusion criteria:

-Those who suffer from other mental illnesses (screen by DSM-V criteria).

-Those who has history of organic brain disease, a pacemaker, gastrointestinal surgery, or serious health problems.

-Those who has history of seizures.

-Those complicated with severe physical disease.

-Those with drug dependence, including alcohol dependence (exception for nicotine dependence).

-Those who took drugs affecting intestinal flora 30 days before and during admission.

-Those who has participated in any other alcohol-related studies or trials within the past

30 days.

-Those who use any prescription or over-the-counter drugs in the past 30 days that may

affect mood or alcohol cravings.

**Sample size estimation**

A total number of 108 patients are needed, with n=36 per arm inclusive of a possible 20% dropout (total n=90; n=30 per arm, without dropout). The sample size was calculated for a parallel group study design involving three arms and was based on power design analysis. This is a higher number required for the primary outcome of AD, and types of interventions as described below. All these calculations were based on the need for a continuous response variable from independent control and experimental subjects, with a ratio of control to subject fixed at 1:1, power of 0.80 and Type-I error probability associated with this test of null hypothesis of 0.05.

Based on the use of ACT for reducing alcohol craving in patients with alcohol use disorder (AUD) and comorbid affective disorder, a sample size of 21 patients per arm is needed, comprising of 26 patients per arm including an additional 20% dropout. The administration of ACT showed decreased OCDS score with a standard deviation of 4.6 within group was observed, accompanied by decreased score of 4.0 between ACT and control groups[42].

Based on the use of probiotics for reducing alcohol dependence syptom in AD patients, a sample size of 30 patients per arm is needed, comprising of 36 patients per arm including an additional 20% dropout. The administration of probiotics showed decreased HAMD score with a standard deviation of 19.25 within group was observed, accompanied by decreased score of 17.75 between probiotics and control groups[43]. Hence, the estimated number of healthy subjects needed for initial comparison with AD subjects was also at 108 subjects.

**Sampling method and subject recruitment (randomization and blinding)**

A single-blind randomized controlled trial design has been chosen. Randomization for the parallel prevention phase will be carried out after checking the inclusion and exclusion criteria. The eligible AD patients will be randomized 1:1:1 ratio to the three arms of the study according to a computer generated, blocked randomization list. The eligible patients will be assigned to the probiotics group, ACT group, and control group with a treatment code concealed in a closed envelope. The randomization will be performed by the study statistician, who had no contact with the patients and not involve in the research project. The statistician is trained to randomize subjects who first enrol in the study into the three groups using computer system for randomization purpose. The allocation sequence will not be available to any member of the research team until databases had been completed and locked. Data collection will be performed by research assistants who were not involved in the study and do not know the objectives of the study. While data analysis will be carried out by the statisticians not involved in the study. Hence, this study is blinded to the researchers but not blinded for the subjects as the ACT group has no parallel control group.

**Data collection and research tools**

In this study, patients with alcohol dependence were given oral probiotics on the basis of conventional treatment. Stool samples and blood samples were collected before and after medication, and the changes of intestinal flora and blood biochemical indexes were analyzed and compared. The EEG changes before and after medication were detected by ERP, and the patients were evaluated regularly with Hamilton Anxiety Scale, Hamilton Depression Scale, alcohol withdrawal Symptom Scale, Pennsylvania Alcohol Desire Scale, etc. To investigate the therapeutic effect of probiotics on patients with alcohol dependence, the changes of intestinal flora and EEG in patients with alcohol dependence. Regular follow-up was performed outside the hospital. The recurrence rate after treatment was analyzed to determine the long-term efficacy of the treatment plan and provide reference and basis for clinical treatment.

***Test drug and treatment plan***

Test drug: Probiotic powder, specification: 2g:0.1g (Probionic Corp., South Korea.).

Placebo drug: Maltodextrin, Specification: 2g:0g (Probionic Corp., South Korea.).

The probiotics, placebo and medications for conventional treatment will be provided by the 2^nd^ Affiliated Hospital of XXMU. There are no other sponsors. The test drug contained both probiotics and prebiotics, and both the test drug and the placebo drug were light yellow powder with no difference in taste. The probiotic powder used in the test drug was maltodextrin as the carrier, which was the same as the placebo drug. Both investigational and placebo drugs should be kept in the temperature range of less than 30 ° C as recommended by the manufacturer.

Treatment: In addition to conventional drug therapy for alcohol dependence, the following interventions were applied to each group:

Routine treatment: alcohol detoxification treatment was carried out at the early stage of admission, mainly with benzodiazepine replacement therapy, while adequate supplementation of B vitamins, strengthening symptomatic supportive treatment, and corresponding antipsychotic drugs, antidepressants or emotional stabilizers were given according to the condition. Benzodiazepines are gradually discontinued after withdrawal symptoms disappear (2 weeks).

Probiotics + conventional drug therapy group: on the basis of conventional drug therapy, probiotics powder 2g, mixed into water or milk, dissolved and oral.

Placebo + conventional drug treatment group: on the basis of conventional drug treatment, maltodextrin of the same weight as the test drug treatment group was mixed into water or milk and orally dissolved.

Frequency of probiotics and placebo treatment: once a day, each time 2g, 8 weeks.

ACT treatment group: Enrolled AD patients will be treated under the ACT protocol for eight weeks. Once a week, each treatment lasted about 1 hour.

***Fecal collection***

Gut microbiota profiles are determined via fecal microbiota analysis. Prior to fecal sample collection, an early announcement will be made one or two weeks earlier and all subjects will be given the fecal collection kit. Materials provided for fecal collection, are fecal collection tube (with RNAlater™ solution and 4 glass beads in tube) and rice paper. A piece of rice paper is floated on water surface in a lavatory bowl (Picture 1). Using the spatula attached to the tube cover, two spatula portions of the feces defecated on the rice paper are collected. Fecal specimens should not be contaminated with water, urine, barium, or mineral oil (Picture 2). Spatula is put back into the fecal collection tube and capped tightly (Picture 3). Then put into ziplock bag, sealed, stored in -80℃ (if possible, can be quick-frozen liquid nitrogen). Fecal sample collection must be completed within one week, upon receiving the fecal collection tube and rice paper. A picture instruction leaflet will be provided prior to fecal collection.

***Questionnaires***

(1) Socio-demographic questionnaire (Chinese version):

This questionnaire covers includes age, marital status, employment, education, monthly income, race, religion, history of medical illnesses, history of psychiatric illness, and history of medication intake, duration of alcohol intake, Average frequency of alcohol intake, average quantity of alcohol intake, and time of last alcohol intake prior to assessment

(2) AUDIT (Chinese version, Figure 1):

This questionnaire can screen drinkers from mild to severe.The reliability and validity of the Chinese version are 0.782. Factor analysis showed that indeed the Chinese version of the AUDIT comprised of three factors and had good convergent and discriminant validity [44]. The scale consists of 10 questions, of which three relate to the amount and frequency of alcohol consumption, three relate to alcohol dependence and four involve in various problems caused by alcohol. The score of the scale ≥ 8 is positive. In general, those with high scores on the first three questions but low scores on the rest suggest serious harmful drinking; High scores in questions 4, 5 and 6 indicate alcohol dependence; High scores in the final section indicate that drinking has caused harm.

(3) CIWA-Ar (Chinese version, Figure 2):

The scale is a standard tool for quantifying the severity of alcohol withdrawal symptoms. The reliability of the Chinese version of CIWA-Ar was good with Cronbach’s α of 0.83 [45]. The total score < 10 suggests mild withdrawal reaction. The score of 10 to 20 indicates moderate. And the total score of more than 20 is considered severe. A severe total score is associated with a risk of delirium tremens and seizures.

(4) VAS (Chinese version, Figure 3):

The VAS has been demonstrated to be accurate for assessment of pain among Chinese patients [46]. It is a simple scale with single factor and single item. A horizontal line corresponding to 0 to 10 points represents the degree of craving from mild to severe. "0" indicates that you don't want to drink at all, and "10" indicates that you really want to drink and cannot restrain yourself. The subjects will be asked to mark the lines according to their perception of themselves.

(5) PACS (Chinese version, Figure 4):

This questionnaire consists of five items to evaluate the severity of craving, including frequency, intensity, duration, difficulty of coping, and average craving degree.The reliability of the Chinese version of PACS was good at Cronbach’s α of 0.97 [47]. It is a seven-point scale from 0 to 6, with 0 being none and 6 being extremely severe. The subjects will be asked to answer questions according to the situation in the past week.

(6) HAMA & HAMD (Chinese version, Figure 5):

These two scales are used to assess the emotional aspects of patients.The reliability of the Chinese version of the HAMD was acceptable with Cronbach’s α of 0.714 [48]. While, the reliability of the Chinese version of the HAMA was 0.93 [49]. HAMA consists of 14 items. The total score ≥ 29 indicates that there may be serious anxiety; The total score ≥ 21 indicates that there must be obvious anxiety; The total score ≥ 14 indicates that there must be anxiety; And the total score ≥ 7 indicates that there may be anxiety. If < 7 points, it is considered that there are no anxiety symptoms. The HAMD we choose is the 24-item version. The total score > 35 indicates severe depression; The total score from 20 to 35 indicates definite depression; And total score from 8 to 20 indicates possible depression. If < 8 points, it is considered normal.

**Figure 1 Figure 2**

**Figure 3 Figure 4**

**Figure 5**

***ERP detection***

(1) Experimental paradigm:

Visual stimuli will be presented by E-Prime 2.0 software. The paradigm consists of three types of images such as i) images related to alcohol cues (common drinking environments, habitual drinking products, etc.), ii) neutral images unrelated to alcohol cues, and iii) task-related images requiring key operation. We will collect EEG while patients watch the stimulation paradigm (Figure 6).

(2) EEG signal acquisition:

We will use the Brain Amp MR-32 instrument to collect signals, and record data through the Brian Vision Recorder software. 64 standard scalp positions will be recorded according to the 10-10 standard lead system (Figure 7). The sampling rate is 1000 Hz, and the impedance between electrode and skin should be less than 5 kΩ.

(3) ERP analysis:

Data will be analyzed using Brian Vision Analyzer 2.1 software. The process includes ① re-reference, ② filtering, ③ removal of ocular artifacts, ④ artifact removal, ⑤ segmentation, ⑥ baseline correction, and ⑦ peak detection.

**Figure 6 Figure 7**

***Blood collection and analyses***

Blood samples collected will be sent to commercial pathology laboratories for pathological profiling which includes red blood cell count, white blood cell counts and platelets count. Serum samples will be analyzed for the interleukins such as interleukin TNF- α , IL-1β, and IL-6 by commercial enzyme-linked immunoabsorbent assay (ELISA) kit following manufacturer’s instructions. Samples may need to be diluted with appropriate amounts of buffer provided in the kit to give constant dilutions prior to analyses. A standard curve will be constructed, and the concentration of samples will be read against the standard curve and expressed as concentration per unit of the samples. The modulation of these immunological parameters will also be determined using gene expression analyses using via real-time PCR.

**Operational definition**

(1) Alcohol dependence: a chronic disease in which a person craves drinks that contain alcohol and is unable to control his or her drinking.

(2) Alcohol withdrawal: a set of symptoms that can occur following a reduction in alcohol use after a period of excessive use. Symptoms typically include anxiety, shakiness, sweating, vomiting, fast heart rate, and a mild fever. More severe symptoms may include seizures, hallucinations, and delirium tremens. Symptoms typically begin around six hours following the last drink, are worst at 24 to 72 hours, and improve by seven days.

(3) Probiotics: Probiotics are live microorganisms promoted with claims that they provide health benefits when consumed, generally by improving or restoring the gut flora.

(4) Acceptance and commitment therapy: a third-generation cognitive behavioral approach which uses acceptance and mindfulness processes, and commitment and behavior change processes to produce psychological flexibility. The latter is defined as the ability to contact the present moment more fully as a conscious human being and to change or persist in behavior when doing so serves valued ends.

(5) Alcohol craving: an intense, urgent, or abnormal desire or longing to consume alcohol.

**Study flowchart**

**Figure 3.1. Flow chart of the study**

**Data analysis**

All data analysis will be performed using SPSS software version 25.0 (SPSS Inc, Chicago, IL, USA). Descriptive statistics for socio-demographic and clinical characteristics, CIWA-Ar, PACS, AUDIT, VAS, HAMD, and HAMA scores, ERP analysis, and serum pro-inflammatory cytokine levels will be computed. All categorical variables will be presented in frequency and percentage, while all continuous variables will be reported in mean and standard deviation or median and interquartile range, depends on normality. Differences in socio-demographic and clinical characteristics between the three groups will be computed with Pearson’s chi square test or one way ANOVA or Kruskal Wallis test.

The mean difference in the primary outcome (CIWA-Ar, PACS, AUDIT, and VAS scores) for the three randomized groups (ACT, probiotic, and palcebo groups) at each specific time point (pre-intervention, 2 weeks at completion of conventional treatment [t0], post-treatment at 8 weeks [t1], post-treatment at 12 weeks [t2], and post-treatment at 24 weeks [t3]) will be assessed using one way analysis of variance (ANOVA) followed by false discovery rate adjustment. For the main pool analysis, mixed ANOVA is used to determine the interaction between the groups (ACT, probiotic, and placebo groups) and time points (t0, t1, t2, and t3) on the primary outcome ( CIWA-Ar, PACS, AUDIT, and VAS scores; interaction = intervention × time; where time is a within-subject variable and intervention effect is a between-subject variable). The main effects of intervention in the groups and time points will be presented as estimated marginal mean and standard error of mean. The study’s primary analysis will follow the intention-to-treat (ITT) principle. The data analysis for the study’s secondary outcomes (severity of anxiety and depressive symptoms, pro-inflammatory cytokines, EEG characteristics, and fecal microbiota) will be conducted similarly to the primary outcomes’ calculation. Statistical significance will be two-tailed and set to p < 0.05.

To handle any missing data, if the missing data represent less than 5% of the study’s total collected data, they will be ignored. If the missing data represent more than 5% but less than 40% of the total collected and are assumed to be randomly missing, then multiple imputation (restricted maximum likelihood estimation) will be performed using Stata 15. However, if the missing data represent more than 40% of the total collected data or are assumed to be missing either not randomly or completely randomly, then only the collected data will be used for the study’s analysis, and the missing data will be explained as a research limitation in any publications of the study’s findings [Jakobsen et al., 2017].

**Expected result(s)**

Table 1. Socio-demographic and clinical characteristics

| Variables | VRET group | | ACT group | | Treatment-as-usual control group | |
| --- | --- | --- | --- | --- | --- | --- |
|  | Frequency (n) | Percentage (%) | Frequency (n) | Percentage (%) | Frequency (n) | Percentage (%) |
| Age* |  |  |  |  |  |  |
| Race:  Han Chinese  Non-Han Chinese |  |  |  |  |  |  |
| Religion:  No religion  Buddhist/Confucianist/Taoist  Other religions |  |  |  |  |  |  |
| Marital status:  Married  Single  Divorce/separated/widow/widower |  |  |  |  |  |  |
| Education:  Up to primary education or lower  Up to secondary education  Up to tertiary education |  |  |  |  |  |  |
| Employment:  Employed  Unemployed/housewife/student  Retired |  |  |  |  |  |  |
| Monthly household income:  < RMB 5000  RMB 5000 to RM 10000  > RMB 10000 |  |  |  |  |  |  |
| History of pre-existing medical illness:  Yes  No |  |  |  |  |  |  |
| History of pre-existing psychiatric illness:  Yes  No |  |  |  |  |  |  |
| History of regular medication intake:  Yes  No |  |  |  |  |  |  |
| Duration of alcohol intake* |  |  |  |  |  |  |
| Average frequency of alcohol intake in a day (times/day)* |  |  |  |  |  |  |
| Average quantity of alcohol intake in a glass (glasses/day)* |  |  |  |  |  |  |
| Last alcohol intake prior to assessment* |  |  |  |  |  |  |

* in mean and standard deviation

Table 2. Comparison of the changes in the Alcohol Use Disorders Identification Test (AUDIT), Clinical Institute Withdrawal Assessment-Alcohol, Revised (CIWA-Ar), Visual Analogue Scale (VAS), and Penn Alcohol Craving Scale (PACS), Hamilton Depression Rating Scale (HAMD), Hamilton Anxiety Rating Scale (HAMA), liver function test variables and γ-glutamyltransferase between those in the probiotics group, acceptance and commitment therapy group, and treatment-as-usual control group and across three timepoints

| **Variables** | **Time 1 (pre-intervention)** | | | **Time 2 (12 weeks)** | | | **Time 3 (24 weeks)** | | | **p-value** | **Effect size** |
| --- | --- | --- | --- | --- | --- | --- | --- | --- | --- | --- | --- |
|  | Pro | ACT | Con | Pro | ACT | Con | Pro | ACT | Con |  |  |
| CIWA-Ar |  |  |  |  |  |  |  |  |  |  |  |
| PACS |  |  |  |  |  |  |  |  |  |  |  |
| AUDIT |  |  |  |  |  |  |  |  |  |  |  |
| VAS |  |  |  |  |  |  |  |  |  |  |  |
| HAMD |  |  |  |  |  |  |  |  |  |  |  |
| HAMA |  |  |  |  |  |  |  |  |  |  |  |
| IL-1β |  |  |  |  |  |  |  |  |  |  |  |
| IL-6 |  |  |  |  |  |  |  |  |  |  |  |
| TNF-α |  |  |  |  |  |  |  |  |  |  |  |
| Fecal microbiota level |  |  |  |  |  |  |  |  |  |  |  |

* statistical significance at p < 0.05, Pro = probiotics group, ACT = acceptance and commitment group and Con = control group

**Gantt chart & milestone**

| **Research activities** | **2023** | **2024** | | **2025** | | **2026** | |
| --- | --- | --- | --- | --- | --- | --- | --- |
|  | **Jul-Dec** | **Jan-Jun** | **Jul-Dec** | **Jan-Jun** | **Jul-Dec** | **Jan-Jun** | **Jul-Dec** |
| Ethical application and approval |  |  |  |  |  |  |  |
| Data collection |  |  |  |  |  |  |  |
| Data analysis and interpretation |  |  |  |  |  |  |  |
| Thesis write-up and submission |  |  |  |  |  |  |  |

| 1. Ethical application and approval completed by December 2023 |
| --- |
| 1. Data collection completed by June 2025. |
| 1. Data analysis and interpretation completed by December 2025 |
| 1. Thesis write-up and submission completed by December 2026 |

**Budget proposal [from the Open Project of Psychiatry and Neuroscience Discipline of Second Affiliated Hospital of Xinxiang Medical University (2022-xyefykfkt-003)]:**

| **Vote**  *Vot* | **Budget Details & Justification**  *Perincian Bajet & Justifikasi* | **Amount requested by researcher (RM)**  *Amaun yang dipohon oleh penyelidik (RM)* | | |
| --- | --- | --- | --- | --- |
|  |  | **Year 1**  *Tahun 1* | **Year 2**  *Tahun 2* | **Year 3**  *Tahun 3* |
| **11000**  Salary and wages  *Gaji dan upah* |  |  |  |  |
| **21000**  Travelling expenses  and subsistence  *Perbelanjaan Perjalanan dan Sara Hidup* |  |  |  |  |
| **23000**  Communication and Utilities (Phone,Fax, Postage etc)  *Perhubungan dan Utiliti*  *(Tel,Faks,Pos, dll)* |  |  |  |  |
| **24000**  Rental  *Sewaan* |  |  |  |  |
| **27000**  Research Materials & Supplies (including Animals, Plants, Disposables, etc.)  *Bekalan & Bahan-bahan Lain (termasuk Haiwan, Pokok dan Bahan Pakai Habis)* | Stationary:  • A4 Paper (5 rims) @ RM12 per rim: RM12 x 6 rims= RM 72 | 24 | 24 |  |
| **28000**  Maintenance and Minor Repair Services  *Penyelenggaraan dan Pembaikan Kecil* |  |  |  |  |
| **29000**  Professional services & other services (printing & hospitality, honorarium, conference fee, journals page charges)  *Perkhidmatan ikhtisas dan perkhidmatan lain-lain (percetakan hospitaliti, honorarium, yuran persidangan & penerbitan)* | (1) Printing:  Photostat copy of questionnaires: at RM0.10 per page x 30 pages x 108 subjects = RM 324 per assessment x 4 assessments = RM 1296  (2) Honorarium of subjects:  RM 20 per subject x 108 subjects x 4 assessments =  RM 10,560 | 1,296  8,640 |  |  |
| **35000 (> RM1,000)**  Equipment[Please provide quotation]  *Peralatan [Sila berikan sebutharga]* |  |  |  |  |
| **TOTAL AMOUNT** | | 9,960 | 24 |  |
| **GRAND AMOUNT** | | **RM 9,984** | | |

**Ethical consideration(s) [if applicable]:**

1. **Subject vulnerability**

Some of the questions asked in the study may cause some subjects to be disturbed. All subjects developed any mental disturbances while participating in the study, they will be recommended for referrals to Henan Mental Hospital, Henan, China (nearest psychiatric service available). In addition, prior to referral, rescue medication such as benzodiazepine will be administered to calm down patients. In addition, subjects will be provided with counselling services from Henan Mental Hospital, Henan, China if needs arise during the study when they experienced mental disturbances. All subjects will be assured anonymity of personal information when they are offered to participate in the study and assured that all the benefits which they were entitled to will be provided if they decided to withdraw from the study. Finally, the subjects will be recommended for referral to support group, such as Alcoholics Anonymous once they have completed the study.

Specific risk may also occur in different groups of alcohol dependent subjects:

1. those who are unemployed may also present with financial constrain and difficulty to get a job. We may recommend for referral to social workers for financial aid and for job manager and occupational therapists in the community psychiatry team in the Department of Psychiatry, 2^nd^ Affiliated Hospital, XXMU for assistance in seeking the right job and for job training.
2. those who have family members may place the close family members at risk of domestic violence and various psychosocial issues. Hence, we may offer help to family members for mental health screening and referrals to counsellors or Department of Psychiatry, 2nd Affiliated Hospital, XXMU for further management.
3. those who are having marital issues with spouse may be recommended for marital counselling under the Department of Psychiatry, 2nd Affiliated Hospital, XXMU.
4. those in the placebo group may be at risk of relapse and experience mental disturbances along the course of the study. Rescue medication such as benzodiazepine may be administered to relieve withdrawal symptoms and if you opt to withdraw from the study, immediate referral to Department of Psychiatry, 2^nd^ Affiliated Hospital, XXMU for further treatment will be carried out.

Subjects may also withdraw from the study should an adverse event occurs. Adverse event (AE) is any untoward medical occurrence in a subject administered a trial intervention that does not necessarily have a causal relationship with this treatment. An AE can be any unfavorable and unintended sign, symptom, or disease temporarily associated with the use of investigational intervention, whether or not related to the investigational intervention. Subjects will be issued with a study card with the contact details of the research team and they are encouraged to maintain close contact by phone, to report any AE occurring in subjects. If the case of adverse event (AE), the event is reported in the adverse event section in the Case report form (CRF) and serious adverse event report is filled if necessary (Figure 5). The details to be reported include the name of the event, date of onset and date of recovery, severity, relationship to the study treatment, measures taken regarding study treatment, treatment of adverse event and outcome of the event (resolved/ongoing). Some reasons that may lead to subjects withdrawing from the study in cases of AE are:

● Presence of adverse reactions not related to the study, but subjects feel uncomfortable to continue this study.

● Presence of adverse reactions that may be related to the study, such as unusual illnesses which started upon this intervention.

● Unusual changes in behavior, temperament, routine, etc of subjects, which started upon this intervention.

● Any suspected or unexpected adverse events that is not consistent with the general acceptance of the ACT and MBSR administration. In general, psychotherapy should not exhibit any side effects or health detrimental effects.

`

**Occurrence of an adverse event (Unexpected or expected)**

**Report to investigators**

**Documentation by investigators:**

- Fill in Case Report Form (CRF)
- If necessary, fill in Serious Adverse Event Report (death, hospitalization)

**Investigators report to sponsor (within 24 hours)**

**Case assessment by sponsor group**

**SUSAR (Suspected Unexpected Serious Adverse Report)**

**Non-SUSAR**

**Report to ethics committee**

**Clinical Trial Database**

**Clinical Study Report**

**Figure 8. Flow chart of adverse event report**

`

1. **Declaration of absence of conflict of interest**

There is no conflict of interest for the research team.

1. **Privacy and confidentiality**

Subjects’ personal identifiable information will not be elicited and they are assured of their participation anonymity. Each subject will be given research number eg RCT001, etc. All the documents involved in assessment of all subjects including subject’s personal information (socio-demographic, substance history, symptomatology, and response to questionnaires) are kept in document files and locked in a cabinet with the key kept by the primary investigator. Only the primary investigator and co-researchers are allowed to access the files for data analysis and for publication purposes. The files will be kept for duration of 7 years after completion of the study and then it will be destroyed completely. The biological specimen (blood and feces) will be discarded after analysis is completed.

1. **Community sensitivities and benefits**

The community will also benefit from the study findings as we provide scientific evidence regarding the effects of two interventions i.e. ACT and probiotics on the alcohol dependence of alcohol dependent patients.

The subjects may benefit from the study as we provide information regarding the mental health of the subjects and any subjects found to have depression and anxiety disorders will also be recommended for referrals to Henan Mental Hospital, Henan, China for further treatment and follow up.

1. **Honorarium and incentives**

As a token of appreciation, respondents will be compensated with honorarium of RMB 200 for each assessment for their willingness and time spent on the study. Therefore, duration of each subject participation in the study is only 30 minutes in each assessment and total duration of participation is 2 hours (sum duration of all 4 assessments).

1. **Other ethical review board approval [if applicable]**

We have obtained approval for the study by the Human Research Ethics Committee of Xinxiang Medical University, Henan, China (code: XYEFYLL-(科研)-2022-28).

1. **Collaborative study terms**

Subject recruitment, data collection and data analysis will be performed in 2^nd^ Affiliated Hospital of XXMU with Dr. Mohammad Farris (USM) providing supervision of the project. Data sharing and publications of study findings will be the responsibility of both AMDI, USM and 2^nd^ Affiliated Hospital of XXMU.

**References**

[1] World Health Organization (WHO). Global Status Report on Alcohol and Health

2018. Geneva: WHO; 2018.

[2] Lim, S. S., Vos, T., Flaxman, A. D., Danaei, G., Shibuya, K., Adair-Rohani, H.,

Amann, M., Anderson, H. R., Andrews, K. G., Aryee, M., Atkinson, C., Bacchus, L.

J., Bahalim, A. N., Balakrishnan, K., Balmes, J., Barker-Collo, S., Baxter, A., Bell,

M. L., Blore, J. D., Blyth, F., … Memish, Z. A. A comparative risk assessment of

burden of disease and injury attributable to 67 risk factors and risk factor clusters in

21 regions, 1990-2010: a systematic analysis for the Global Burden of Disease

Study 2010. Lancet 2012; 380(9859): 2224–2260.

[3] Deng, H., Zhang, B., Wang, C. A review of EEG-based electrophysiological

techniques for diagnosing and treating alcohol dependence. Chin J Drug Depend

2021; 30(2): 92–96.

[4] Hao, W. Guideline for diagnosis and treatment of alcohol-related disorders.

Beijing: People's Medical Publishing House; 2014.

[5] Eşel, E., Dinç, K. Neurobiology of Alcohol Dependence and Implications on

Treatment. Turk Psikiyatri Derg 2017; 28(1): 51–60.

[6] Parker, C. C., Lusk, R., Saba, L. M. Alcohol Sensitivity as an Endophenotype of

Alcohol Use Disorder: Exploring Its Translational Utility between Rodents and

Humans. Brain Sci 2020; 10(10): 725.

[7] Ma, H., Zhu, G. The dopamine system and alcohol dependence. Shanghai Arch

Psychiatry 2014; 26(2): 61–68.

[8] Ohgi Y. Alcohol dependence and opioid receptor- pharmacological profile of

nalmefene Nihon Yakurigaku Zasshi 2020; 155(3): 145–148.

[9] Mykhaylyshyn U. В. Social- psychological features of value orientations of patients

with alcohol dependence. Wiad Lek 2019; 72(4): 579–583.

[10]Ibáñez, M. I., Camacho, L., Mezquita, L., Villa, H., Moya-Higueras, J., Ortet, G.

Alcohol Expectancies Mediate and Moderate the Associations between Big Five

Personality Traits and Adolescent Alcohol Consumption and Alcohol-Related

Problems. Front Psychol 2015; 6: 1838.

[11]Fairbairn, C. E., Sayette, M. A. A social-attributional analysis of alcohol response.

Psychol Bull 2014; 140(5): 1361–1382.

[12]Mutlu, E.A.; Gillevet, P.M.; Rangwala, H.; Sikaroodi, M.; Naqvi, A.; Engen, P.A.;

Kwasny, M.; Lau, C.K.;Keshavarzian, A. Colonic microbiome is altered in

alcoholism. Am. J. Physiol. Gastrointest. Liver Physiol.2012, 302, G966–G978.

[13]Bajaj, J.S.; Ridlon, J.M.; Hylemon, P.B.; Thacker, L.R.; Heuman, D.M.; Smith, S.;

Sikaroodi, M.; Gillevet, P.M.Linkage of gut microbiome with cognition in hepatic

encephalopathy. Am. J. Physiol. Gastrointest. Liver Physiol. 2012, 302, G168 –

G175.

[14]Han, S.H.; Suk, K.T.; Kim, D.J.; Kim, M.Y.; Baik, S.K.; Kim, Y.D.; Cheon, G.J.;

Choi, D.H.; Ham, Y.L.; Shin, D.H. Effects of probiotics (cultured Lactobacillus

subtilis/Streptococcus faecium) in the treatment of alcoholic hepatitis: Randomizedcontrolled

multicenter study. Eur. J. Gastroenterol. Hepatol. 2015, 27, 1300–1306.

[15]Leclercq, S.; Matamoros, S.; Cani, P.D.; Neyrinck, A.M.; Jamar, F.; Stärkel, P.;

Windey, K.; Tremaroli, V.; Bäckhed, F.; Verbeke, K. Intestinal permeability, gutbacterial

dysbiosis, and behavioral markers of alcohol-dependence severity. Proc.

Natl. Acad. Sci. USA 2014, 111, E4485–E4493.

[16]Kirpich, I.A.; Solovieva, N.V.; Leikhter, S.N.; Shidakova, N.A.; Lebedeva, O.V.;

Sidorov, P.I.; Bazhukova, T.A.; Soloviev, A.G.; Barve, S.S.; McClain, C.J.

Probiotics restore bowel flora and improve liver enzymes in human alcohol-induced

liver injury: A pilot study. Alcohol 2008, 42, 675–682.

[17]Keshavarzian, A.; Choudhary, S.; Holmes, E.W.; Yong, S.; Banan, A.; Jakate, S.;

Fields, J.Z. Preventing gut leakiness by oats supplementation ameliorates alcoholinduced

liver damage in rats. J. Pharmacol. Exp. Ther. 2001, 299, 442–448.

[18]Ferrier, L.; Bérard, F.; Debrauwer, L.; Chabo, C.; Langella, P.; Buéno, L.;

Fioramonti, J. Impairment of the intestinal barrier by ethanol involves enteric

microflora and mast cell activation in rodents. Am. J. Pathol.2006, 168, 1148–1154.

[19]Forsyth, C.B.; Farhadi, A.; Jakate, S.M.; Tang, Y.; Shaikh, M.; Keshavarzian, A.

Lactobacillus GG treatment ameliorates alcohol-induced intestinal oxidative stress,

gut leakiness, and liver injury in a rat model of alcoholic steatohepatitis. Alcohol

2009, 43, 163–172.

[20]Xie, G.; Zhong, W.; Zheng, X.; Li, Q.; Qiu, Y.; Li, H.; Chen, H.; Zhou, Z.; Jia, W.

Chronic ethanol consumption alters mammalian gastrointestinal content metabolites.

J. Proteome Res. 2013, 12, 3297–3306.

[21]Bull-Otterson, L.; Feng, W.; Kirpich, I.; Wang, Y.; Qin, X.; Liu, Y.; Gobejishvili,

L.; Joshi-Barve, S.; Ayvaz, T.; Petrosino, J. Metagenomic analyses of alcohol

induced pathogenic alterations in the intestinal microbiome and the effect of

Lactobacillus rhamnosus GG treatment. PLoS ONE 2013, 8, e53028.

[22]Leclercq, S.; De Saeger, C.; Delzenne, N.; de Timary, P.; Stärkel, P. Role of

inflammatory pathways, blood mononuclear cells, and gut-derived bacterial

products in alcohol dependence. Biol. Psychiatry 2014, 76,725–733.

[23]Hasin, D.S.; Stinson, F.S.; Ogburn, E.; Grant, B.F. Prevalence, correlates, disability,

and comorbidity of DSM-IV alcohol abuse and dependence in the United States:

Results from the National Epidemiologic Survey on Alcohol and Related

Conditions. Arch. Gen. Psychiatry 2007, 64, 830–842.

[24]Nanji, A. A., Khettry, U. & Sadrzadeh, S. M. Lactobacillus feeding reduces

endotoxemia and severity of experimental alcoholic liver (disease).

Proc.Soc.Exp.Biol.Med. 1995(205):243–247.

[25]Kirpich, I. A. et al. Probiotics restore bowel flora and improve liver enzymes in

human alcohol-induced liver injury: a pilot study. Alcohol 2008(42):675–682 .

[26]Stadlbauer, V. et al. Effect of probiotic treatment on deranged neutrophil function

and cytokine responses in patients with compensated alcoholic cirrhosis. J. Hepatol.

2008(48): 945–951 (2008).

[27]Loguercio, C. et al. Beneficial effects of a probiotic VSL#3 on parameters of liver

dysfunction in chronic liver diseases. J. Clin. Gastroenterol. 2005(39):540 – 543

(2005).

[28]Tillisch, K. et al. Consumption of fermented milk product with probiotic modulates

brain activity. Gastroenterology 2013(144):1394–1401, 1401–4 .

[29]Savignac, H. M. et al. Prebiotic feeding elevates central brain derived neurotrophic

factor, N-methyl-D-aspartate receptor subunits and D-serine. Neurochem. Int.

2013(63):756–764 .

[30]Li, X. & Wolf, M. E. Multiple faces of BDNF in cocaine addiction. Behav. Brain

Res.2015(279): 240–254 .

[31]Temko, J. E. et al. The Microbiota, the Gut and the Brain in Eating and Alcohol

Use Disorders: A ‘Ménage à Trois’? Alcohol Alcohol 2017(52):403–413 .

[32]Hayes, S. C., Strosahl, K. D., Wilson, K. G. Acceptance and commitment therapy:

An experiential approach to behaviour change. The Guilford Press: New York,

1999.

[33]Johns, S. A., Stutz, P. V., Talib, T. L., Cohee, A. A., Beck-Coon, K. A., Brown, L.

F., Wilhelm, L. R., Monahan, P. O., LaPradd, M. L., Champion, V. L., Miller, K.

D., Giesler, R. B. Acceptance and commitment therapy for breast cancer survivors

with fear of cancer recurrence: a 3-arm pilot randomized controlled trial. Cancer

2019;126:211-218.

[34]Hayes, S. C., Luoma, J. B., Bond, F. W., Masuda, A., Lillis, J. Acceptance and

commitment therapy: model, processes and outcomes. Behaviour Research and

Therapy 2006;44(1):1–25.

[35]Henry, J. L., Wilson, P. H., Bruce, D. G., Chisholm, D. J., Rawling, P. J. Cognitivebehavioural

stress management for patients with noninsulin dependent diabetes

mellitus. Psychology, Health & Medicine 1997;2(2):109-118.

[36]Mumtaz W, Vuong PL, Malik AS, et al. A review on EEG-based methods for

screening and diagnosing alcohol use disorder[J]. Cogn Neurodyn, 2018, 12(2):

141-156.

[37]Euser AS, Arends LR, Evans BE, et al. The P300 event-related brain potential as a

neurobiological endophenotype for substance use disorders: a meta-analytic

investigation[J]. Neurosci Biobehav Rev, 2012, 36(1): 572-603.

[38]Bartholow BD, Lust SA, Tragesser SL. Specificity of P3 event-related potential

reactivity to alcohol cues in individuals low in alcohol sensitivity[J]. Psychol

Addict Behav, 2010, 24(2): 220-228.

[39]Petit G, Kornreich C, Verbanck P, et al. Gender differences in reactivity to alcohol

cues in binge drinkers: a preliminary assessment of event-related potentials[J].

Psychiatry Res, 2013, 209(3): 494-503.

[40]Field M, Munafò MR, Franken IH. A meta-analytic investigation of the relationship

between attentional bias and subjective craving in substance abuse[J]. Psychol Bull,

2009, 135(4): 589-607.

[41]Deng, H., Zhang, B., Du, K., Hao, W., Wang, C. An event-related potentials study

of alcohol craving state evoked by visual cues. Chin J Nerv Ment Dis 2021; 47(04):

208–214.

[42] Thekiso, T. B., Murphy, P., Milnes, J., Lambe, K., Curtin, A., Farren, C. K.

Acceptance and Commitment Therapy in the Treatment of Alcohol Use Disorder

and Comorbid Affective Disorder: A Pilot Matched Control Trial. Behav Ther 2015;

46(6): 717–728.

[43] Ghorbani Z, Nazari S, Etesam F, Nourimajd S, Ahmadpanah M, Jahromi SR. The

effect of synbiotic as an adjuvant therapy to fluoxetine in moderate depression: a

randomized multicenter trial. Arch Neurosci. (2018) 5:e60507. doi:

10.5812/archneurosci.60507.

[44] Cong Zhang,Guoping Yang,Zhen Li,et al.Reliability and validity of the Chinese version on Alcohol Use Disorders Identification Test[J].Chinese Journal of Epidemiology.2017,38(8):1064-1067.

[45] Chuanjun Zhuo,Yueqin Huang,Jitao Li,et al. Validity and reliability of Chinese version of Clinic Alcohol Withdrawal Syndrome Scale.Chinese Mental Health Journal.2010,24(5):347-350,374.

[46] Aun C, Lam YM, Collett B. Evaluation of the use of visual analogue scale in Chinese patients. Pain. 1986;25(2):215-221.

[47] Wei Wang,Jisheng Tang,Binglun Liu.The reliability and Validity of Penn Alcohol Craving Scale(PACS) [J].Journal of Psychiatry.2010,23(1):29-30.

[48] Zheng Y, Zhao J, Philips M, Liu J, Cai M, Sun S, Huang M. Validity and reliability of the Chinese version of the Hamilton Depression Rating Scale. British Journal of Psychiatry. 1988;152:660-664.

[49] Wang, C., Chu, Y., Zhang, Y., Zhang, N., Zhang, J., and Yang, H. (2011). Study on factor structure of hamilton rating scale for anxiety. J. Clin. Psychiatry. 2011;21:299–301.
