# Supplementary material for: Research protocol of the efficacy of probiotics for the treatment of alcohol use disorder among adult males: A comparison with placebo and acceptance and commitment therapy in a randomized controlled trial
Source: PLoS One. 2023 Dec 5;18(12):e0294768. doi: 10.1371/journal.pone.0294768 (PMC10697511; doi:10.1371/journal.pone.0294768)
Supplement: S2 Appendix — (DOC) [file pone.0294768.s002.doc]

**Additional file 2. Participant information sheet and consent form for this study (English version)**

JAWATANKUASA ETIKA PENYELIDIKAN (MANUSIA) – JEPeM USM

UNIVERSITI SAINS MALAYSIA

RESEARCH INFORMATION (TRANSLATED ENGLISH COPY)

Research Title: The Efficacy of Probiotics for the Treatment of Alcohol Use Disorder among Adult Males: A comparison with Placebo and Acceptance and Commitment Therapy (For AUD subjects)

*Name of main and co-Researcher: Dr. Mohammad Farris Iman Leong Bin Abdullah (MMC: 43103), Zhang Bingyu, Nurul Izzah Shari*

#### INTRODUCTION

You are invited to take part voluntarily in an interventional research. This research is about comparing the efficacy of acceptance and commitment therapy (ACT; a psychotherapy technique) and probiotics in reducing alcohol craving and treating alcohol dependence, normalizing the the characteristics of electroencephalogram (EEG; a measuring device connected to the brain to measure the electrical activities of the brain) under various alcohol cue and blood pro-inflammatory cytokines (chemicals in blood which is elevelated when one has alcohol dependence, depression, and anxiety), and reducing severity of depression and anxiety.

It is important that you read and understand this research information before agreeing to participate in this study. You will receive a copy of this form to keep for your records if you agree to participate.

Your participation in this study is expected to take a total duration of 2 hours (30 minutes per assessment and there are 4 assessments) in a period of 24 weeks to complete. This study is estimated to include up to 120 participants.

#### PURPOSE OF THE STUDY

The purpose of this study are to compare the efficacy of acceptance and commitment therapy (ACT; a psychotherapy technique) and probiotics in reducing alcohol craving and treating alcohol dependence, normalizing the the characteristics of electroencephalogram (EEG; a measuring device connected to the brain to measure the electrical activities of the brain) under various alcohol cue and blood pro-inflammatory cytokines (chemicals in blood which is elevelated when one has alcohol dependence, depression, and anxiety), and reducing severity of depression and anxiety.

#### PARTICIPANTS CRITERIA

The research team members will discussed your eligibility to participate in this study. It is important that you are completely truthful with the staff including your health history.

This study will include individual (alcohol dependence patients) who are:

-Hospitalized patients diagnosed with alcohol use disorder

-Male, age 18 to 55 years old, Han nationality, junior high school education or above,

right-handed.

-Those with normal eyesight (including corrected vision).

This study will not incude individual (alcohol dependence patients) who are:

-Patients suffer from other mental illnesses.

-Patients with allergy to the active ingredient or excipients.

-The patient had a history of organic brain disease, a pacemaker, gastrointestinal surgery, or serious health problems.

-The patient had a history of seizures.

-The patient was complicated with severe physical disease.

-Patient has other drug dependence (in addition to nicotine dependence).

-The patient took drugs affecting intestinal flora 30 days before and during admission.

-The patient has participated in any other alcohol-related studies or trials within the past

30 days.

-Patients had use any prescription or over-the-counter drugs in the past 30 days that may

affect mood or alcohol cravings.

STUDY PROCEDURES

All participants will be given treatment-as-usual, such as benzodiazepine replacement therapy, low-dose antipsychotics, and adequate vitamin B. Then, participants with 2 weeks of abstinence from alcohol. will be randomized into three groups i.e. acceptance and commitment therapy (ACT), vitual reality exposure therapy (VRET), and treatment-as-usual control group. Participants are then assessed with Alcohol Use Disorders Identification Test (AUDIT), Clinical Institute Withdrawal Assessment-Alcohol, Revised (CIWA-Ar), Visual Analogue Scale (VAS), Penn Alcohol Craving Scale (PACS), with emotional rating scales, such as Hamilton Anxiety Scale (HAMA), and Hamilton Depression Scale (HAMD), blood collection for assessing pro-inflammatory cytokines (IL-1β, IL-6, TNF-α), fecal sampling for fecal microbiota analysis. Participants will also undergo event-related potential (ERP). Assessments will be carried out at four timelines i.e. at baseline, 2 weeks after intervention, 8 weeks after baseline (immediately after completion of intervention), and 24 weeks after baseline assessment.

(1) In ERP assessment, you will be attached to the Brain Amp MR-32 instrument to collect signals, and record data through the Brian Vision Recorder software. 64 standard scalp positions will be recorded (as in the figures below). The sampling rate is 1000 Hz, and the impedance between electrode and skin should be less than 5 kΩ. Visual stimuli will be presented such as i) images related to alcohol cues (common drinking environments, habitual drinking products, etc.), ii) neutral images unrelated to alcohol cues, and iii) task-related images requiring key operation. We will collect EEG while patients watch the stimulation paradigm.

(2) Acceptance and commitment therapy (ACT): It is a third-generation cognitive behavioral approach which uses acceptance and mindfulness processes, and commitment and behavior change processes to produce psychological flexibility. Unlike CBT, which aims to change unhelpful thoughts and feelings, ACT was designed to increase adaptive coping through acceptance, cognitive defusion, mindfulness, and perspective-taking exercises while supporting cancer survivors in aligning behavior with their personal values. Hence, ACT consists of 8 sessions, one session per week to facilitate cancer patients to development and maintenance of health behavioral improvements by targeting internal barriers, such as emotional discomfort and self-defeating thoughts, and by fostering connection and commitment to personal values associated with self-management of positive health behaviors.

(3) Probiotic *Lactobacillus* sp.: For intake of the probiotic Lactobacillus sp. or placebo, you will be given an 8-week supply of fixed Lactobacillus sp. Sachets (9 log CFU/day) or placebo. The Lactobacillus sp. Probiotic or the placebo powder can be consumed directly from the sachet or mixed it with a drink or cold water, and it can be consumed at any time during the day. The intake stops after 8 weeks, and you will be monitored for another 16 weeks. Subsequent visits will occur at week 0, 2, 8 and 24.

Feces and blood sample collection:

The feces and blood samples will be collected at week 0, 2, 8, and 24. The first sample will be collected before you started consumed the beneficial Lactobacillus sp. And the placebo (at the week before the intervention starts) or the ACT starts. At week 8, the supplement intake and ACT ends and you will be monitored for another 16 weeks. We will collect the last sample at week-24.

The feces specimen will be collected in a small quantity in a clean and dry container. The feces specimen will not be contaminated with water, urine, barium or oily mineral. You would be required to transfer the feces specimen from the collection container to the transportation bottle by using spork at the closing cap from the fecal sample transportation vial. You are required to collect sufficient fecal quantity by filling the volume to the “filled until here” line of the transportation vial and then close the sealer tightly and share the content sufficiently for mixing it. The blood sample will be collected as suggested by the doctor in the research team. After you have collected the samples, we will obtain the fecal sample from you and the blood sample will be given to the doctors in the research team.

There will be no genetic test or collection of any familial genetic information in this study.

RISKS

Participation in this research has minimal risk. Nevertheless, if you experience emotional disturbance after answering the questionnaires, we will recommend reference to counsellor in Advanced Medical and Dental Institute, Universiti Sains Malaysia and the 2nd Affiliated Hospital of Xinxiang Medical University, Henan, China. If you still exhibit depressive and anxiety symptoms after you have completed the study, we will recommend reference to Advanced Medical and Dental Institute, Universiti Sains Malaysia and the Department of Psychiatry, 2nd Affiliated Hospital of Xinxiang Medical University, Henan, China for further assessment and treatment as necessary. Please inform the research team if you encounter any problems or if you have any important information which will change your participation in the study.

In addition, prior to referral, rescue medication such as benzodiazepine will be administered to calm down patients. In addition, subjects will be provided with counselling services from Advanced Medical and Dental Institute, Universiti Sains Malaysia and Henan Mental Hospital, Henan, China if needs arise during the study when they experienced mental disturbances. All subjects will be assured anonymity of personal information when they are offered to participate in the study and assured that all the benefits which they were entitled to will be provided if they decided to withdraw from the study. Finally, the subjects will be recommended for referral to support group, such as Alcoholics Anonymous once they have completed the study.

Specific risk may also occur in different groups of alcohol dependent subjects:

(a) those who are unemployed may also present with financial constrain and difficulty to get a job. We may recommend for referral to social workers for financial aid and for job manager and occupational therapists in the community psychiatry team in Advanced Medical and Dental Institute, Universiti Sains Malaysia and the Department of Psychiatry, 2nd Affiliated Hospital, XXMU for assistance in seeking the right job and for job training.

(b) those who have family members may place the close family members at risk of domestic violence and various psychosocial issues. Hence, we may offer help to family members for mental health screening and referrals to counsellors or Advanced Medical and Dental Institute, Universiti Sains Malaysia and the Department of Psychiatry, 2nd Affiliated Hospital, XXMU for further management.

(c) those who are having marital issues with spouse may be recommended for marital counselling under Advanced Medical and Dental Institute, Universiti Sains Malaysia and the Department of Psychiatry, 2nd Affiliated Hospital, XXMU.

(d) those in the placebo group may be at risk of relapse and experience mental disturbances along the course of the study. Rescue medication such as benzodiazepine may be administered to relieve withdrawal symptoms and if you opt to withdraw from the study, immediate referral to Advanced Medical and Dental Institute, Universiti Sains Malaysia and the Department of Psychiatry, 2nd Affiliated Hospital, XXMU for further treatment will be carried out.

REPORTING HEALTH EXPERIENCES.

Please contact, at any time, the following researcher if you experience any health problem either directly or indirectly related to this study.

Dr. Mohammad Farris Iman Leong Bin Abdullah [MMC Registration No._43103___] at +604-5622482 or +6018-6669950.

#### PARTICIPATION IN THE STUDY

Your taking part in this study is entirely voluntary. You may refuse to take part in the study or you may stop your participation in the study at anytime, without any penalty or loss of benefits to which you are otherwise entitled. Your participation also may be stopped by the research team without your consent if in any form you have violated the study eligibility criteria. The research team member will discussed with you if the matter arises.

#### POSSIBLE BENEFITS [Benefit to Individual, Community, University]

The research procedures will be provided to you without any cost. You will received information regarding your mental health status which are expected to be important to safeguard your mental health after you were diagnosed with alcohol use disorder. You will also enrolled in intervention sessions in acceptance and commitment therapy (ACT) or probiotics intake which is expected to help you to maintain your mental health.

The findings of this study will hopefully bring benefits to the community by providing evidence that the two psychotherapy intervention will alleviate alcohol dependence and prevent relapse. This will allowed recommendation to integrate these two interventions into the treatment regime of alcohol addiction as data on the efficacy of these two interventions among patients with alcohol use disorder is scarce.

You will not recieve any compensation from this study. There will be no insurance for participating in this study, but if there is any study-related injury or disability involving the participants and family members, the treatment and rehabilitation expenses will be covered fully by the research team. However you may get reimbursement for your travelling cost while in the study duration. This study does not plan to develop any commercial product from its findings.

#### QUESTIONS

If you have any question about this study or your rights, please contact;

Dr. Mohammad Farris Iman Leong Bin Abdullah

Primary investigator

Department of Community Health

Advanced Medical and Dental Institute

Universiti Sains Malaysia

SAINS@BERTAM

13200 Kepala Batas

Pulau Pinang

Malaysia

+6018-6669950

Zhang Bingyu

Co-primary investigator

Department of Community Health

Advanced Medical and Dental Institute

Universiti Sains Malaysia

SAINS@BERTAM

13200 Kepala Batas

Pulau Pinang

Malaysia

(+86) 176-3070-0996

If you have any questions regarding the Ethical Approval or any issue / problem related to this study, please contact;

Mr. Mohd Bazlan Hafidz Mukrim

Secretary of Human Research Ethics Committee USM

Division of Research & Innovation (R&I)

USM Health Campus

Tel. No. : +609-767 2354 / +609-767 2362

Email : [bazlan@usm.my](mailto:bazlan@usm.my)

OR

Miss Nor Amira Khurshid Ahmed

Secretariat of Human Research Ethics Committee USM

Research Creativity & Management Office (RCMO)

USM Main Campus, Penang

Tel. No. : +604-6536537

Email : [noramira@usm.my](mailto:noramira@usm.my)

The investigators serve only as investigator of this study and they are not the one who provide service to the participants.

#### CONFIDENTIALITY

Your information will be kept confidential by the researchers and will not be made publicly available unless disclosure is required by law.

Data obtained from this study that does not identify you individually will be published for knowledge purposes.

Your original records may be reviewed by the researcher, the Ethical Review Board for this study, and regulatory authorities for the purpose of verifying the study procedures and/or data. Your information may be held and processed on a computer. Only research team members are authorized to access your information. The information will be stored for 2 years after completion of the study before it is discarded following standard procedures. While any biological sample (blood and feces) will be discarded following standard procedures once analysis is completed in the study. Any future possible use of the data and specimen collected for research purpose will be communicated to you by the research team and you may refuse to consent for future use and we will discard the data and specimen accrodingly.

The feedback on the study findings will be informed by the research team after completion of the study upon request from the participants.

By signing this consent form, you authorize the record review, information storage and data process described above.

#### SIGNATURES

To be entered into the study, you or a legal representative must sign and data the signature page [ATTACHMENT S or or ATTACHMENT P]

ATTACHMENT S

Subject Information and Consent Form

(Signature Page)

Research Title: The Efficacy of Probiotics for the Treatment of Alcohol Use Disorder among Adult Males: A comparison with Placebo and Acceptance and Commitment Therapy (For AD subjects)

*Name of main and co-Researcher: Dr. Mohammad Farris Iman Leong Bin Abdullah (MMC: 43103), Zhang Bingyu, Nurul Izzah Shari*

To become a part this study, you or your legal representative must sign this page. By signing this page, I am confirming the following:

- I have read all of the information in this Patient Information and Consent Form including any information regarding the risk in this study and I have had time to think about it.
- All of my questions have been answered to my satisfaction.
- I voluntarily agree to be part of this research study, to follow the study procedures, and to provide necessary information to the doctor, nurses, or other staff members, as requested.
- I may freely choose to stop being a part of this study at anytime.
- I have received a copy of this Participant Information and Consent Form to keep for myself.

Participant Name

Participant I.C No

Signature of Participant or Legal Representative Date (dd/MM/yy)

Name of Individual

Conducting Consent Discussion

Signature of Individual Date (dd/MM/yy)

Conducting Consent Discussion

Name & Signature of Witness Date (dd/MM/yy)

Note: i) All participants who are involved in this study will not be covered by insurance.

ATTACHMENT P

Participant’s Material Publication Consent Form

Signature Page

Research Title: The Efficacy of Probiotics for the Treatment of Alcohol Use Disorder among Adult Males: A comparison with Placebo and Acceptance and Commitment Therapy (For AD subjects)

*Name of main and co-Researcher: Dr. Mohammad Farris Iman Leong Bin Abdullah (MMC: 43103), Zhang Bingyu, Nurul Izzah Shari*

To become a part this study, you or your legal representative must sign this page.

By signing this page, I am confirming the following:

- I understood that my name will not appear on the materials published and there have been efforts to make sure that the privacy of my name is kept confidential although the confidentiality is not completely guaranteed due to unexpected circumstances.
- I have read the materials or general description of what the material contains and reviewed all photographs and figures in which I am included that could be published.
- I have been offered the opportunity to read the manuscript and to see all materials in which I am included, but have waived my right to do so.
- All the published materials will be shared among the medical practitioners, scientists and journalist world wide.
- The materials will also be used in local publications, book publications and accessed by many local and international doctors world wide.
- I hereby agree and allow the materials to be used in other publications required by other publishers with these conditions:
- The materials will not be used as advertisement purposes nor as packaging materials.
- The materials will not be used out of contex – i.e.: Sample pictures will not be used in an article which is unrelated subject to the picture.

Participant Name

Participant I.C No. Participant’s Signature Date (dd/MM/yy)

Name and Signature of Individual Date (dd/MM/yy)

Conducting Consent Discussion

Note: i) All participants who are involved in this study will not be covered by insurance.

**JAWATANKUASA ETIKA PENYELIDIKAN (MANUSIA) – JEPeM USM**

**UNIVERSITI SAINS MALAYSIA**

**RESEARCH INFORMATION (TRANSLATED ENGLISH COPY)**

**Research Title: The Efficacy of Probiotics for the Treatment of Alcohol Use Disorder among Adult Males: A comparison with Placebo and Acceptance and Commitment Therapy (For healthy subjects)**

***Name of main and co-Researcher: Dr. Mohammad Farris Iman Leong Bin Abdullah (MMC: 43103), Zhang Bingyu, Nurul Izzah Shari***

#### **INTRODUCTION**

You are invited to take part voluntarily in an interventional research. This research is about comparing the efficacy of acceptance and commitment therapy (ACT; a psychotherapy technique) and probiotics in reducing alcohol craving and treating alcohol dependence, normalizing the the characteristics of electroencephalogram (EEG; a measuring device connected to the brain to measure the electrical activities of the brain) under various alcohol cue and blood pro-inflammatory cytokines (chemicals in blood which is elevelated when one has alcohol dependence, depression, and anxiety), and reducing severity of depression and anxiety.

It is important that you read and understand this research information before agreeing to participate in this study. You will receive a copy of this form to keep for your records if you agree to participate.

Your participation in this study is expected to take about 30 minutes to complete. This study is estimated to include up to 120 participants.

#### **PURPOSE OF THE STUDY**

The purpose of this study are to compare the efficacy of acceptance and commitment therapy (ACT; a psychotherapy technique) and probiotics in reducing alcohol craving and treating alcohol dependence, normalizing the the characteristics of electroencephalogram (EEG; a measuring device connected to the brain to measure the electrical activities of the brain) under various alcohol cue and blood pro-inflammatory cytokines (chemicals in blood which is elevelated when one has alcohol dependence, depression, and anxiety), and reducing severity of depression and anxiety.

#### **PARTICIPANTS CRITERIA**

The research team members will discussed your eligibility to participate in this study. It is important that you are completely truthful with the staff including your health history.

This study will include individual (healthy controls) who are:

-Male, age 18 to 55 years old, Han nationality, junior high school education or above,

right-handed.

-No history of psychoactive substance abuse (except tobacco).

-Those with normal eyesight (including corrected vision).

-No alcoholic beverages in the last 2 weeks.

-According to the WHO healthy alcohol consumption standard, the average intake of

pure alcohol per week is less than 210 grams, or do not drink at all.

This study will not incude individual (healthy controls) who are:

-Patients suffer from other mental illnesses.

-Patients with allergy to the active ingredient or excipients.

-The patient had a history of organic brain disease, a pacemaker, gastrointestinal surgery, or serious health problems.

-The patient had a history of seizures.

-The patient was complicated with severe physical disease.

-Patient has other drug dependence (in addition to nicotine dependence).

-The patient took drugs affecting intestinal flora 30 days before and during admission.

-The patient has participated in any other alcohol-related studies or trials within the past

30 days.

-Patients had use any prescription or over-the-counter drugs in the past 30 days that may

affect mood or alcohol cravings.

**STUDY PROCEDURES**

All healthy subjects will undergo blood collection for assessing pro-inflammatory cytokines (IL-1β, IL-6, TNF-α), fecal sampling for fecal microbiota analysis and EEG signal acquisition through the use of the Brain Amp MR-32 instrument to collect signals, and record data through the Brian Vision Recorder software. 64 standard scalp positions will be recorded according to the 10-10 standard lead system (in the figures below). The sampling rate is 1000 Hz, and the impedance between electrode and skin should be less than 5 kΩ. Assessment is only conducted once.

Feces and blood sample collection:

The feces and blood samples will be collected only once after you have enrolled in the study. The feces specimen will be collected in a small quantity in a clean and dry container. The feces specimen will not be contaminated with water, urine, barium or oily mineral. You would be required to transfer the feces specimen from the collection container to the transportation bottle by using spork at the closing cap from the fecal sample transportation vial. You are required to collect sufficient fecal quantity by filling the volume to the “filled until here” line of the transportation vial and then close the sealer tightly and share the content sufficiently for mixing it. The blood sample will be collected as suggested by the doctor in the research team. After you have collected the samples, we will obtain the fecal sample from you and the blood sample will be given to the doctors in the research team.

There will be no genetic test or collection of any familial genetic information in this study.

**RISKS**

Participation in this research has minimal risk. Nevertheless, if you experience emotional disturbance after enrolled in the study, we will recommend reference to counsellor in Advanced Medical and Dental Institute, Universiti Sains Malaysia and the 2nd Affiliated Hospital of Xinxiang Medical University, Henan, China. Please inform the research team if you encounter any problems or if you have any important information which will change your participation in the study.

**REPORTING HEALTH EXPERIENCES.**

Please contact, at any time, the following researcher if you experience any health problem either directly or indirectly related to this study.

Dr. Mohammad Farris Iman Leong Bin Abdullah **[MMC Registration No._43103___]** at +604-5622482 or +6018-6669950.

#### **PARTICIPATION IN THE STUDY**

Your taking part in this study is entirely voluntary. You may refuse to take part in the study or you may stop your participation in the study at anytime, without any penalty or loss of benefits to which you are otherwise entitled. Your participation also may be stopped by the research team without your consent if in any form you have violated the study eligibility criteria. The research team member will discussed with you if the matter arises.

#### **POSSIBLE BENEFITS [Benefit to Individual, Community, University]**

The research procedures will be provided to you without any cost. You will received information regarding your mental health status.

The findings of this study will hopefully bring benefits to the community by providing evidence that the two psychotherapy intervention will alleviate alcohol dependence and prevent relapse. This will allowed recommendation to integrate these two interventions into the treatment regime of alcohol addiction as data on the efficacy of these two interventions among patients with alcohol use disorder is scarce.

You will not receive any compensation from this study. There will be no insurance for participating in this study, but if there is any study-related injury or disability involving the participants and family members, the treatment and rehabilitation expenses will be covered fully by the research team. However you may get reimbursement for your travelling cost while in the study duration.

This study does not plan to develop any commercial product from its findings.

#### **QUESTIONS**

If you have any question about this study or your rights, please contact;

**Dr. Mohammad Farris Iman Leong Bin Abdullah**

**Primary investigator**

**Department of Community Health**

**Advanced Medical and Dental Institute**

**Universiti Sains Malaysia**

**SAINS@BERTAM**

**13200 Kepala Batas**

**Pulau Pinang**

**Malaysia**

**+6018-6669950**

**Zhang Bingyu**

**Co-primary investigator**

**Department of Community Health**

**Advanced Medical and Dental Institute**

**Universiti Sains Malaysia**

**SAINS@BERTAM**

**13200 Kepala Batas**

**Pulau Pinang**

**Malaysia**

**(+86) 176-3070-0996**

If you have any questions regarding the Ethical Approval or any issue / problem related to this study, please contact;

**Mr. Mohd Bazlan Hafidz Mukrim**

**Secretary of Human Research Ethics Committee USM**

**Division of Research & Innovation (R&I)**

**USM Health Campus**

**Tel. No. : +609-767 2354 / +609-767 2362**

**Email :** [**bazlan@usm.my**](mailto:bazlan@usm.my)

**OR**

**Miss Nor Amira Khurshid Ahmed**

**Secretariat of Human Research Ethics Committee USM**

**Research Creativity & Management Office (RCMO)**

**USM Main Campus, Penang**

**Tel. No. : +604-6536537**

**Email :** [**noramira@usm.my**](mailto:noramira@usm.my)

The investigators serve only as investigator of this study and they are not the one who provide service to the participants.

#### **CONFIDENTIALITY**

Your information will be kept confidential by the researchers and will not be made publicly available unless disclosure is required by law.

Data obtained from this study that does not identify you individually will be published for knowledge purposes.

Your original records may be reviewed by the researcher, the Ethical Review Board for this study, and regulatory authorities for the purpose of verifying the study procedures and/or data. Your information may be held and processed on a computer. Only research team members are authorized to access your information.

The information will be stored for 2 years after completion of the study before it is discarded following standard procedures. While any biological sample (blood and feces) will be discarded following standard procedures once analysis is completed in the study. Any future possible use of the data and specimen collected for research purpose will be communicated to you by the research team and you may refuse to consent for future use and we will discard the data and specimen accrodingly.

The feedback on the study findings will be informed by the research team after completion of the study upon request from the participants.

By signing this consent form, you authorize the record review, information storage and data process described above.

#### **SIGNATURES**

To be entered into the study, you or a legal representative must sign and data the signature page **[ATTACHMENT S or or ATTACHMENT P]**

**ATTACHMENT S**

**Subject Information and Consent Form**

**(Signature Page)**

**Research Title: The Efficacy of Probiotics for the Treatment of Alcohol Use Disorder among Adult Males: A comparison with Placebo and Acceptance and Commitment Therapy (For healthy subjects)**

***Name of main and co-Researcher: Dr. Mohammad Farris Iman Leong Bin Abdullah (MMC: 43103), Zhang Bingyu, Nurul Izzah Shari***

To become a part this study, you or your legal representative must sign this page. By signing this page, I am confirming the following:

- I have read all of the information in this Patient Information and Consent Form **including any information regarding the risk in this study** and I have had time to think about it.
- All of my questions have been answered to my satisfaction.
- I voluntarily agree to be part of this research study, to follow the study procedures, and to provide necessary information to the doctor, nurses, or other staff members, as requested.
- I may freely choose to stop being a part of this study at anytime.
- I have received a copy of this Participant Information and Consent Form to keep for myself.

**Participant Name**

**Participant I.C No**

**Signature of Participant** or Legal Representative **Date** (dd/MM/yy)

**Name of Individual**

Conducting Consent Discussion

**Signature of Individual** **Date** (dd/MM/yy)

Conducting Consent Discussion

**Name & Signature of Witness** **Date** (dd/MM/yy)

Note: i) All participants who are involved in this study will not be covered by insurance.

**ATTACHMENT P**

**Participant’s Material Publication Consent Form**

**Signature Page**

**Research Title: The Efficacy of Probiotics for the Treatment of Alcohol Use Disorder among Adult Males: A comparison with Placebo and Acceptance and Commitment Therapy (For healthy subjects)**

***Name of main and co-Researcher: Dr. Mohammad Farris Iman Leong Bin Abdullah (MMC: 43103), Zhang Bingyu, Nurul Izzah Shari***

To become a part this study, you or your legal representative must sign this page.

By signing this page, I am confirming the following:

- I understood that my name will not appear on the materials published and there have been efforts to make sure that the privacy of my name is kept confidential although the confidentiality is not completely guaranteed due to unexpected circumstances.
- I have read the materials or general description of what the material contains and reviewed all photographs and figures in which I am included that could be published.
- I have been offered the opportunity to read the manuscript and to see all materials in which I am included, but have waived my right to do so.
- All the published materials will be shared among the medical practitioners, scientists and journalist world wide.
- The materials will also be used in local publications, book publications and accessed by many local and international doctors world wide.
- I hereby agree and allow the materials to be used in other publications required by other publishers with these conditions:
- The materials will not be used as advertisement purposes nor as packaging materials.
- The materials will not be used out of contex – i.e.: Sample pictures will not be used in an article which is unrelated subject to the picture.

**Participant Name**

**Participant I.C No. Participant’s Signature** **Date** (dd/MM/yy)

**Name and Signature of Individual** **Date** (dd/MM/yy)

Conducting Consent Discussion

Note: i) All participants who are involved in this study will not be covered by insurance.
