# Supplementary material for: Research protocol of the efficacy of probiotics for the treatment of alcohol use disorder among adult males: A comparison with placebo and acceptance and commitment therapy in a randomized controlled trial
Source: PLoS One. 2023 Dec 5;18(12):e0294768. doi: 10.1371/journal.pone.0294768 (PMC10697511; doi:10.1371/journal.pone.0294768)
Supplement: S3 Appendix — (DOCX) [file pone.0294768.s003.docx]

**Additional file 4. Summary of the ACT module in this study:**

*Session 1:* This session will be started with developing a therapeutic relationship and an initial intake interview with respondents. The personal information of respondents such as the main external barrier, fusion past, experiential avoidance, unworkable action regarding alcohol use, strengths, resources and past life history will be gathered. Based on the information given, the therapist will generate a case formulation. Then, the case formulation will be used in mapping the respondents' life issues on a matric diagram.

*Session 2:* The therapist will start the session by discussing the issues from the previous session and homework. Then, the respondents will be encouraged to embrace their unpleasant feelings and thoughts (creative hopelessness) which arise from the urge to drink, the symptoms of AUD, and multiple failed attempts to remain sober. The therapist will introduce the new possibilities such as the unpleasant feelings and thoughts are not the enemy, but control is the problem. The alternative to control is a willingness to give space and “sit” with the unpleasant thought, feeling, and memory. Then, the respondents are encouraged to actively “contact” with their psychological experiences without struggling, “sit with” and make space for their experiences and remain at the present moment. Several metaphors will be used such as tug of war with the monster and physicalizing the unpleasant thoughts and feelings. The metaphors will help the respondents to recognize their experiential avoidance and “let go” of unhelpful emotion-control strategies.

*Session 3:* In this session, the therapist will conduct a defusion session with the respondents. Defusion will help to reduce behavioural avoidance from unpleasant emotions and thoughts. Defusion is aimed to increase the extent to which respondents’ abilities to choose actions based on their values rather than being stuck and controlled by their emotions and thoughts. The therapist will use metaphors such as hands trap, the mind is a bully and passengers on the bus and performs pushing paper exercise with respondents. As for homework, the therapist will encourage respondents to do physicalizing exercises and passengers on the bus when they deal with their unwanted thoughts and feelings.

*Session 4:* In this session, the therapist will guide the respondents to practice structured mindfulness exercises namely mindful breathing. In the mindfulness exercise, the therapist will encourage the respondents to focus on the present moment, for example in breathing, the respondents are encouraged to use their senses during breathing by focusing on inhaling and exhaling, noticing the temperature of the air, and movement of their muscles during breath in and out. Then, awareness will be expanded by noticing the external environment such as sights and sounds from her surrounding, sensations of body contact and body posture. After that, the respondents will be asked to do mindful breathing while simultaneously acknowledge the presence of their unpleasant thoughts and feelings. The therapist will encourage the respondents to experience the thoughts and feeling with openness, interest and receptiveness without attempted to change them. Instead of trying to distract respondents from their unpleasant feelings and thoughts, the purpose of this activity was to awake respondents that there are many more things that happened right now that can be appreciated and enjoyed rather than overly focused on unpleasant feelings and thoughts. When the respondents become more aware, they could respond efficiently, particularly when experiencing unpleasant feelings and thoughts. The therapist will use the metaphor of an emotional storm and dropping anchor. In this metaphor, the therapist emphasizes that dropping an anchor does not make the storm leave but it hold the boat firmly during the storm. The storm will come and go on its own. As homework, the respondents will be encouraged to practice mindfulness. At least, mindful breathing twice daily, after wake-up in the morning and before goes to sleep. The respondents also need to record mindfulness exercises in the mindfulness worksheet.

*Session 5:* In this session, the therapist will check the mindfulness exercise worksheet and ask respondents’ experience in practicing mindfulness. Then, the therapist also encouraged the respondent to use mindfulness in her daily activities such as mindful eating and mindful bathing.

*Session 6:* This session is aimed to develop respondents’ sense of self as context. The respondents will learn that the self is safe, continuous, consistent and separate from their unpleasant thoughts and feelings. The respondents will be asked to develop a position from which they can observe their unpleasant thoughts and feelings and let them come and go. Again, the therapist will use metaphors such as chessboard and furnished room to ensure that respondents understand the concept of self as context.

*Session 7:* This session involved therapeutic processes in hexaflex namely values. This session is aimed to ensure that respondents have clearly expressed their values. The therapist will help the respondents to clarify their values and identify what important in their life such as relationships, health, education and spirituality. Values are the anticipated qualities of ongoing action, freely chosen and never need to be evaluated. Values differ from goals. Once values are identified, then, goals can be established. Some exercises such as values assessment, values compass and bull eye were conducted to help the respondents differentiate between values and goals, identify and evaluate whether their life are engaged with their values or not.

*Session 8:* In this session, the therapist will discuss again the values determined by the respondent from the previous session. This session aims to ensure that respondents will be able to link their values to action strategies. The therapist will ask respondents to list the feasible actions and strategies. Finally, the therapist will encourage the respondents to take at least minimal steps or actions that could move in the direction of their valued life.
